# Impact of Solvent Choice on SERS Sensing: Solvent–Analyte and Solvent–Substrate Effects for Organophosphate and Nerve Agent Detection

**DOI:** 10.1021/acs.jpca.6c02753

**Published:** 2026-08-03

**Authors:** Rasmus Öberg, Jonas Segervald, Andreas Larsson, Magnus Andersson, Per Ola Andersson

**Affiliations:** † Swedish Defence Research Agency (FOI), Umeå 90621, Sweden; ‡ Department of Physics, Umeå University, Umeå 90736, Sweden; § Umeå Centre for Microbial Research (UCMR), Umeå 90736, Sweden

## Abstract

Organophosphate chemicals such as herbicides, pesticides, and nerve agents are harmful even in small amounts, thus requiring methods for rapid ultrasensitive detection. Surface-enhanced Raman spectroscopy (SERS) is a leading method for trace-level detection of dissolved chemicals, yet our understanding of the critical influence of the solvent is currently incomplete. Rather than being recognized as a crucial aspect for generating a strong signal enhancement, the solvent is often dismissed as a secondary parameter. This has left the dual impact of solvents on the analyte and SERS substrate properties in relation to SERS largely unexplored. In this work, we evaluated the suitability of common solvents for the detection of nerve agents using SERS on gold nanopillar SERS substrates. Overall, we found large differences in the limit of detection (LOD) between solvents. VX exhibited an LOD of 0.2 ppm in water, compared to 8.7 and 6.3 ppm in ethanol and acetonitrile, respectively, with similar trends for other nerve agents. Dichloromethane and *n*-hexane were found to be largely ineffective as solvents for SERS in these applications. To explain these differences, we used DFT to model analyte–solvent interactions, showing that high-polarity solvents such as water generate higher electron concentrations in select analyte molecular groups, impacting analyte–substrate interactions. Furthermore, we used Raman mapping, contact angle measurements, and SEM to evaluate solvent–substrate interactions, showing that solvent-dependent capillary forces impact SERS substrate nanogeometry and thus the formation of SERS hotspots. Overall, these results provide insight into the many solvent effects that are relevant to designing future SERS sensing assays.

## Graphical abstract

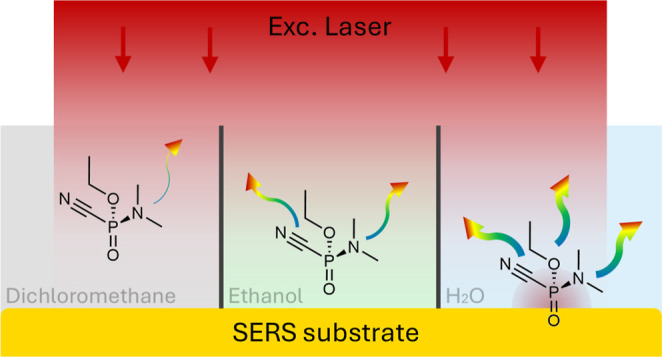

## 1 Introduction

Organophosphates are a group of toxic chemicals that are used for civilian purposes as flame retardants and pesticides but also for military purposes as nerve agents.^1^ By inhibiting acetylcholine breakdown in the body, they overburden the nervous system, which can lead to seizures, respiratory failure, and eventually death.^2,3^ The historic and potential use of nerve agents in warfare and terrorism creates a need for technologies and methods to treat nerve agent poisoning but also methods to detect and prevent nerve agent poisoning before it occurs.^4,5^ Currently, several methods are available in the field of nerve agent sensing. These methods include ion mobility spectroscopy and flame photometry, which, although limited in specificity, are well-suited to indicate the presence of nerve agents.^6^ However, while these methods work well to detect high-volatility nerve agents such as sarin (GB) and soman (GD), they are less suitable for low-volatility nerve agents such as VX, tabun (GA), and cyclosarin (GF), which are less likely to occur in the gas phase under ambient conditions.^7^ Because of this, it is important to develop sensitive detection methods for low-volatility nerve agents in solutions and on surfaces.

One such detection method is Raman spectroscopy. Raman spectroscopy is a vibrational spectroscopic technique that generates a rich and molecule-specific spectral signature, giving it an advantage over less specific sensing techniques, e.g., fluorescence spectroscopy or colorimetric methods. This spectral richness in combination with the ease and speed with which Raman spectroscopy can be performed makes it ideal for detecting and identifying unknown chemicals. However, Raman scattering is a very weak phenomenon, with only approximately 1 in 10,000,000 photon–molecule interactions producing Raman scattering. Because of this, to make Raman detection applicable in real sensing scenarios, e.g., by first responders and in situ investigators, surface-enhanced Raman spectroscopy (SERS) is considered a promising technique.^8^ In SERS, the Raman signal is enhanced by strong induced electromagnetic fields near metal nanostructures, generated by localized surface electrons in the nanostructures oscillating in resonance with an excitation laser. This resonance enhances the electromagnetic field intensity and, thus, the Raman signal intensity of any sample molecules in close proximity to the nanostructured surface. In the last few decades, SERS has seen rapid development and application in fields as diverse as forensics,^9–11^ medicine,^12,13^ environmental sensing,^14^ and most relevant to this work: defense.^15–18^ The growing research into SERS, in combination with the advent of portable and hand-held Raman spectroscopy equipment, has thus paved the way for the rapid and sensitive field detection of many harmful materials.

There are many challenges in developing effective SERS sensing assays, but an often overlooked aspect is the impact of the solvent. Different common solvents, such as water and ethanol, interact differently with various chemicals, e.g., by modifying their dielectric properties.^19,20^ This can in turn impact the intensity of molecular vibrations in the chemicals or change their affinity to the SERS substrate. Furthermore, solvents may directly act on the chemistry and morphology of the SERS substrate. Examples of the latter include capillary forces from a solvent that act to aggregate dispersed nanoparticles or modifying the geometry of a nanostructured SERS surface.^21,22^ One popular geometry for SERS substrate applications uses tightly packed noble metal-coated nanopillars to generate SERS hotspots.^23–25^ These nanopillar structures have previously been shown to aggregate when exposed to the capillary forces of an evaporating solvent droplet, forming highly SERS-active nanopillar aggregates.^22,26^ These solvent effects become even more important to consider in field applications using simpler analyte deposition methods such as drop deposition, where the solvent may not be homogeneously distributed on the SERS-active surface. Together, these factors paint a complex picture of how solvent choice can impact SERS detection.

In this work, we explore the effects of common solvents on a nerve agent SERS detection assay using popular gold-coated nanopillar substrates and a portable Raman system. We measured the SERS activity of nerve agents VX, GA, and GF in five different solvents—deionized (DI) water (H_2_O), ethanol (EtOH), acetonitrile (MeCN), dichloromethane (DCM), and *n*-hexane. These solvents were chosen to represent a range of solvent polarities, as well as organic and inorganic solvents.^27^ The nerve agents were drop-deposited onto nanostructured SERS surfaces, and their SERS spectral characteristics were evaluated. To better understand the impact of the solvent on the physical properties of the nerve-agent molecule, we used density functional theory (DFT) calculations to calculate electron density distributions for different solvation parameters. Furthermore, we evaluated the effects of SERS substrate–solvent interactions using contact angle measurements, scanning electron microscopy (SEM), and Raman mapping on the substrate. Lastly, we discuss practical aspects of choosing a suitable solvent for a detection assay. Although the impact of solvent choice on SERS is well-known, these effects are rarely discussed in full, and even more rarely considered are solvent effects on both the analyte molecule and the SERS substrate. We thus hope that the results herein can provide insight into how to effectively evaluate solvents for future SERS studies on dissolved analytes using nanostructured SERS surfaces.

## 2 Materials and Methods

### 2.1 Nerve Agents and Solvents

The nerve agents VX, GA, and GF used herein were synthesized in house with purity >90%, as confirmed by ^1^H and ^13^C NMR. For solvents, we used Milli-Q DI H_2_O, EtOH (CAS 64-17-5, >99.9%, Sigma-Aldrich), MeCN (CAS 75-05-8, >99.9%, Sigma-Aldrich), DCM (CAS 75-09-2, >99%, Sigma-Aldrich), and *n*-hexane (CAS 110-54-3, >99%, Sigma-Aldrich).

### 2.2 SERS Substrates

In this work, we used 3 × 3 mm silicon surfaces decorated with gold-coated silicon nanopillars as SERS substrates (Au SERSstrate, Silmeco). The nanopillars have a width of ∼50 nm and are placed with a density of ∼33 nanopillars/μm^2^ on the surface. The silicon pillars are 230 nm high and coated in a 160 nm thick Au metal film, making a total pillar height of ∼390 nm.

### 2.3 Raman Systems and Methodology

To evaluate the SERS characteristics of our nerve agent samples, we used a portable self-calibrating miniRaman spectrometer (Lightnovo ApS, Copenhagen) equipped with a 785 nm continuous wave laser. We used a laser power of 8 mW and measured the Raman shifts between 400 and 2500 cm^–1^, with a spectral resolution of 8–10 cm^–1^. The spot size diameter of the laser was approximately 15 μ m. The laser and spectrometer were both controlled using the associated miniRaman Software. Ahead of Raman measurements, the calibration of the instrument was validated using a silicon wafer with a known Raman profile.

Each Raman measurement consisted of ten accumulations with each accumulation lasting two seconds, for a total measurement time of 20 s. For each sample, we performed five separate measurements each on three different SERS substrates for a total of 15 measurement points. The choice to measure the Raman signal from discrete points rather than map the entire substrate was motivated by the application to portable sensing, which often requires portable/hand-held Raman equipment with less-developed mapping capabilities. Raman measurements were performed on nerve agents deposited on SERS substrates at concentrations ranging from 0 to 1000 ppm. First, reference spectra were collected from the pristine SERS substrates, followed by sequential sample depositions and spectral acquisitions from a given nerve agent/solvent combination at concentrations 0, 0.1, 0.3, 1, 3, 10, 30, 100, 300, and 1000 ppm. Unless otherwise declared, the given Raman wavenumbers correspond to those found at the maximum measurable concentration. An averaged SERS spectrum from pristine SERS substrates can be found in Figure S1.

Raman maps were acquired using a separate miniRaman microscope system (Lightnovo ApS, Copenhagen). This Raman microscope operates similarly to the miniRaman spectrometer with a 785 nm continuous wave laser, spot size ∼15 μ m, range 400–2500 cm^–1^, and spectral resolution of ∼8 cm^–1^. Raman mapping was performed on a 30 × 30 point grid of 3 × 3 mm corresponding to the area of the SERS substrates. Each point was measured for 0.5 s, with a laser power of 8 mW. Each mapping was performed in triplicate on three different SERS substrates. Raman maps were measured on 3–300 ppm nerve agent solutions in H_2_O, EtOH, and MeCN.

### 2.4 Data Analysis

We processed our SERS spectra using the OPUS vibrational spectroscopy software (OPUS 8.5 SP1, Bruker Optics GmbH & Co.). The acquired spectra were cut to the range 500-2500 cm^–1^ and baseline-corrected using the concave rubber band method with 64 baseline points and 15 iterations. From these baseline-corrected spectra, we then calculated the average spectra and standard deviations (SD). The SERS band intensity growth was modeled using a Langmuir growth model for ligand binding *f*(*x*) = *Ax* ^*n*^/(*k* ^*n*^ + *x* ^*n*^), where *x* is the analyte concentration, *k* is the analyte concentration producing half site occupation, *n* is the Hill coefficient, and *A* is a proportionality coefficient.^28^ We plotted our spectra using the Origin (2023, OriginLab) software, with each shown spectrum being an average of 15 acquisitions from 3 separate SERS substrates. The error bars represent 2 standard deviations from the average values. We calculated the LOD of the different nerve agents dissolved in the respective solvents as the lowest concentration where the Raman signal from the nerve agent exceeds the blank signal plus 3 standard deviations (SD) of the spectral noise at a given Raman shift wavenumber at 0 ppm, as done in previous work detailing SERS detection.^29,30^

### 2.5 Contact Angle Measurements

To evaluate the surface tension from the solvent on the SERS substrate, we used a Theta OneAttention optical tensiometer (Biolin scientific Oy, Espoo) to measure the contact angle between different solvents and the SERS substrate. We used the live analysis feature of the OneAttension software to continuously measure the contact angle, with a time resolution of 10 measurements/second. The tensiometer was calibrated using a tungsten carbide ball with known dimensions. Using a pipet, we then deposited 2 μL of each solvent on the SERS substrates and measured the contact angle. The contact angle was measured from five separate droplets of each solvent, as well as from both sides of each droplet.

### 2.6 SEM Imaging

We imaged our SERS substrates before and after exposure to the solvents using a scanning electron microscope (SEM) (Merlin FESEM, Carl Zeiss) and collected the SEM images at magnifications ranging from 1000 to 80,000× using the InLens imaging mode. To avoid charge build-up, we applied conductive silver paint (SCP03B, Electrolube) between the SERS substrates and the carbon paper-decorated sample holder before imaging.

### 2.7 DFT Calculations

We used density functional theory to calculate theoretical electron density distributions at the level of theory B3LYP^31–34^ /cc-pvtz.^35,36^ Calculations on binding energies of GA to a Au single-atom structure for molecular groups with similar electrostatic potentials were performed at the B3LYP/LANL2DZ^37^ level of theory. These calculations were performed using Gaussian 16 ver. C.01 software,^38^ with an ultrafine integration grid and implicit solvation in water, ethanol, acetonitrile, dichloromethane, and *n*-hexane describing solvents as continuous dielectric media using the polarizable continuum model.^39^ All molecular geometries were visualized using Gaussview 6 software.

## 3 Results and Discussion

### 3.1 Solvent Effects on SERS Spectra of Nerve Agents

SERS detection of low-volatility nerve agents is traditionally challenging due to their low lethal doses and inherent Raman cross-sections. Consequently, practical detection protocols must be optimized for maximum sensitivity.^40^ One of these optimization factors is the choice of the solvent. Although we herein focus specifically on nerve agents, many of the following considerations regarding solvent–analyte and solvent–substrate interactions remain applicable to a broad range of SERS-active materials. With this in mind, we study the impact of solvent choice on the SERS spectra from nerve agents on common Au nanopillar SERS substrates. We will first present the SERS spectral evolution of different combinations of nerve agents and solvents with a focus on the SERS characteristics of the nerve agents. This will be followed by experiments designed to better understand the analyte–solvent interactions and solvent–substrate interactions that impact the earlier described SERS spectra. For the sake of brevity, we herein elect to limit our discussion to the traditional, identifying Raman bands of each nerve agent molecule as observed both by us and previously in the literature and how the choice of solvent impacts these bands. Additional broader nonorganophosphate bands originating from native SERS substrate structures were consistently present between 800 and 1000, 1100 and 1300, and 1500 and 1700 cm^–1^ (Figure S1).

Starting with VX, we observed a broad SERS band around 900 cm^–1^ using H_2_O, EtOH, and MeCN as solvents (Figure 1A–C), alongside a smaller band around 750 cm^–1^. These bands appear here consistent with previous studies, with the more intense 900 cm^–1^ band having been assigned to the C–C–N and O–P–C stretching modes of VX.^41,42^ We elect to focus our analysis on the prominent 900 cm^–1^ band. We found that solvent choice significantly affected SERS sensitivity; for example, the 900 cm^–1^ band emerged at 0.3 ppm in H_2_O, while EtOH and MeCN required higher concentrations of ∼ 3 ppm. A notable concentration-dependent spectral shift was also observed, where the band centered at 920 cm^–1^ at low concentrations gradually downshifted to 900 cm^–1^ between 10 and 1000 ppm. This phenomenon possibly stems from a change in molecular bond lengths or a shift in the molecular binding geometries relative to the substrate as surface coverage increases and multilayers start to form. A shift in the molecular geometry relative to the SERS substrate could promote different vibration modes for Raman scattering, e.g., the C–C–N band relative to the O–P–C band, causing shifts in the position of the Raman band. Similar shifts have also been observed in previous work detailing the SERS detection of VX, suggesting that the shift observed herein is not an isolated observation.^43^ It should also be noted that the region around the 900 cm^–1^ band was subject to a notable variance in the background signal between substrates (here most clearly visible in Figure 1B), which means that additional care should be taken when using these substrates to detect VX. Similar principles apply to other analyte/substrate combinations whenever there is a significant overlap between the signal and background spectra. Looking at the change in Raman intensity of the 900 cm^–1^ band, we observed a large decrease in band intensity when approaching high VX concentrations >100 ppm. A similar phenomenon has previously been observed on both functionalized and nonfunctionalized SERS substrates in earlier work.^42,44,45^ This effect is likely caused in part by the formation of VX multilayers, which impede the SERS signal because the nonsurface layer molecules experience limited plasmonic enhancement. Although this is a common and expected observation in SERS spectroscopy, it is noteworthy that it is only observed in VX out of the three nerve agents. We believe that this is in part due to the larger molecular size of VX than that of the G-series agents studied herein, as well as the polar nature of the VX molecule (to be discussed in Section 3.2) possibly promoting direct dipole–dipole interactions between molecules, which can in turn encourage multilayer formation.

**Figure 1:**
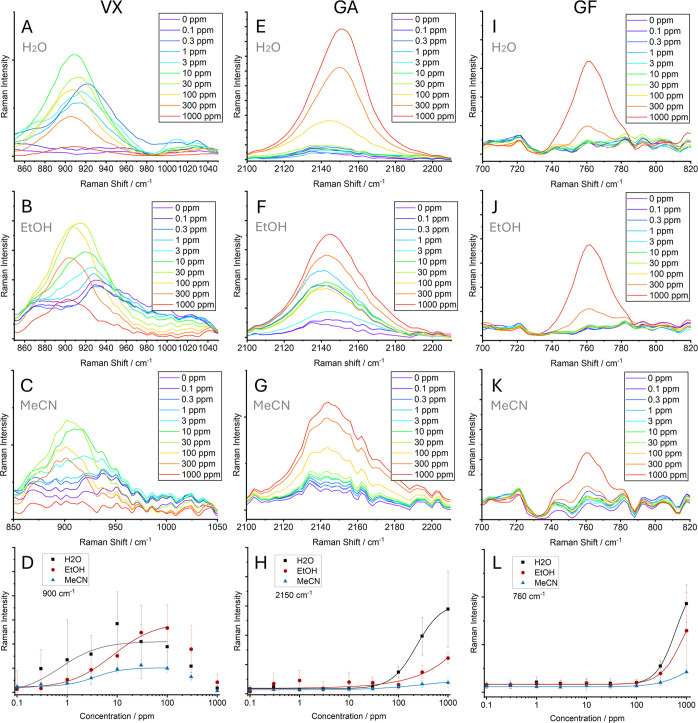
SERS spectral evolutions of VX, GA, and GF in different solvents. Evolutions of the VX 900 cm^–1^ band using H_2_O (A), EtOH (B), and MeCN (C) as solvents are displayed in the respective panels, with the change in SERS intensity summarized in (D). Evolutions of the GA 2150 cm^–1^ band using H_2_O (E), EtOH (F), and MeCN (G) as solvents are displayed in the respective panels, with the change in SERS intensity summarized in (H). Evolutions of the GF 760 cm^–1^ band using H_2_O (I), EtOH (J), and MeCN (K) as solvents are displayed in the respective panels, with the change in SERS intensity summarized in (L). Fitting parameters for panels (D), (H), and (L) are found in Table S1. Note that fitting curves for VX in panel (D) are limited to 100 ppm due to the drop in intensity at higher concentrations.

Looking at the other solvents, we observed qualitatively similar SERS spectral characteristics from VX using EtOH and MeCN as we did from H_2_O. Both EtOH and MeCN gave rise to the same Raman band around 900 cm^–1^ and the same wavenumber downshift over increasing concentrations (Figure 1B,C). However, the intensity of the 900 cm^–1^ band was overall lower when using EtOH and MeCN as solvents compared to H_2_O, especially at lower VX concentrations, resulting in lower signal-to-noise ratios (SNR). This can be seen more clearly when comparing the intensity of the 900 cm^–1^ band at increasing VX concentrations for the different solvents (Figure 1D). The lower SERS intensities observed in EtOH and MeCN compared to H_2_O naturally resulted in a higher LOD. We calculated an LOD of 0.2 ppm for VX in H_2_O, compared to 8.7 and 6.3 ppm in EtOH and MeCN, respectively. These LODs in EtOH and MeCN are in line with recent observations on VX by Zappalà et al. using methanol (MeOH) as a solvent.^42^ This is a reasonable comparison as the chemical similarities between EtOH and MeOH should yield similar SERS characteristics. It should be noted that due to the notable spectral background around 900 cm^–1^, these LOD estimations are susceptible to background interference. As such, it is prudent to be conservative in similar situations when determining LOD, taking into account the spectral and background variance. Interestingly, although the calculated LOD for VX in H_2_O was significantly lower than in EtOH, EtOH noted similar or even higher Raman intensities at higher VX concentrations (>30 ppm). This phenomenon is likely in large part caused by differences in the way the different solvents spread the analyte over the surface and the resulting differences in the analyte surface concentration. These factors are discussed in Section 3.3. Lastly, little to no Raman signal was recorded when using DCM or *n*-hexane as solvents. This will be a common theme between all the nerve agents discussed in this work, and several possible reasons for this observation are discussed in Sections 2.2 and 2.3. Full spectral evolutions of VX in all five solvents can be found in Figures S2–S6.

Moving on to GA, we observed an intense Raman band around 2150 cm^–1^ when using H_2_O, EtOH, and MeCN as solvents (Figure 1E–H), qualitatively similar to what has previously been observed in the literature.^42,43^ This vibrational mode is classically attributed to the C≡N stretching mode originating from the cyanide group of the GA molecule.^46^ Similarly to VX, we could observe a slight wavenumber shift in the 2150 cm^–1^ band from ∼2145 cm^–1^ at low GA concentrations to 2150 cm^–1^ at higher concentrations >30 ppm. Interestingly, unlike the wavenumber shift in VX, this shift cannot be explained by different vibrational modes as the 2150 cm^–1^ is commonly attributed to a single vibration. Instead, this shift could be caused by small changes in bond length in more tightly packed molecular configurations or possibly different binding modes between the GA molecule and SERS substrate. The latter possibility is discussed in more detail in Section 3.2. Comparing the different solvents, we see that H_2_O yields the most pronounced SERS band at 2150 cm^–1^, followed by EtOH and MeCN, in line with what was observed in VX. This is also reflected in the LOD of GA in the different solvents, which were found to be 46, 62, and 75 ppm in H_2_O, EtOH, and MeCN, respectively. These LODs are approximately 5–10× higher than those previously observed on the same analyte on similar substrates by Zappalà et al.^47^ Although the nature behind this discrepancy is difficult to ascertain, it can likely be attributed in part to their use of methanol as a solvent, as well as to the use of a different Raman instrument. Again, little to no spectral GA characteristics were observed when using DCM or *n*-hexane as solvents. Full spectral evolutions of GA in all five solvents can be found in Figures S7–S11.

Compared to VX and GA, GF is widely considered the most difficult nerve agent to detect using SERS of the three studied here, as established in several previous studies.^44,48^ This is in line with what we found in our experiments, with no significant SERS profile observed from GF at concentrations of <100 ppm using any solvent. However, at higher concentrations >100 ppm, we could observe the gradual appearance and growth of a SERS band at 760 cm^–1^ (Figure 1I–L), matching previous observations by Hakonen et al.^48^ This band has previously been assigned by Christensen et al. to the P–C stretching vibration of GF.^49^ Similar to observations in VX and GA, the 760 cm^–1^ band intensities were noticeably higher in H_2_O compared to EtOH or MeCN. Also, similar to the other nerve agents, there was a large relative variance between the measurements, as indicated by the error bars (Figure 1D,H,L), especially when using H_2_O and EtOH as solvents. We calculated the LOD of GF to be 214, 267, and 395 ppm in H_2_O, EtOH, and MeCN, respectively, following the previously observed trend of a lower LOD when using H_2_O as solvent, followed by a higher LOD in EtOH, and a yet higher LOD in MeCN. Once again, little to no spectral GF characteristics were observed using DCM or *n*-hexane as solvents. Full spectral evolutions of GF in all five solvents can be found in Figures S12–S16. The LODs for all combinations of nerve agents and solvents are summarized in Table 1.

**Table 1:** Calculated LOD (ppm) of VX, GA, and GF in Different Solvents H_2_O, EtOH, MeCN, DCM, and *n*-Hexane

Solvent/Nerve agent	VX	GA	GF
H_2_O	0.2	46	214
EtOH	8.7	62	267
MeCN	6.3	75	395
DCM/*n*-hexane	—	—	—

Overall, it is evident from these results that the choice of solvent has a significant impact on the SERS characteristics of different nerve agents. H_2_O consistently yielded higher SERS signals and lower LODs than EtOH, MeCN, DCM, and *n*-hexane. This is consistent with previous observations of Tripathi et al., who found higher SERS intensities when using H_2_O as a solvent for the model molecules thiophenol (TP) and 1,2-bis­(4-pyridyl)-ethylene (BPE) compared to, e.g., EtOH and MeCN.^19^ However, in their experiments, they found that MeCN yielded similar or higher SERS intensities for these molecules when used as a solvent compared with EtOH. Specifically, the SERS signal from TP was noticeably higher when using MeCN as a solvent compared with EtOH, and the SERS intensities from BPE were largely identical between H_2_O/EtOH and H_2_O/MeCN mixtures. Although we prefer to be cautious with direct comparisons between the work by Tripathi et al. and the results herein, since the choice of SERS surface geometry (nanopillar vs inverted pyramid) and the deposition method (drop deposition vs immersion) differs between the two studies, the differences are nonetheless worth discussing. These differences suggest that the impact of solvent choice on SERS detection is caused by a combination of first-order interactions between the analyte and solvent and second-order effects between the solvent and the SERS substrate, impacting interactions with the analyte. Examples of the former include, e.g., dipole–dipole interactions between analyte and solvent, as well as chemical reactions between the two. Meanwhile, examples of the latter might include solvent-induced changes in substrate nanomorphology^22^ and the effects of solvent–substrate interactions on the surface concentration of the analyte.^50^ Thus, we explore these two factors in the following sections.

### 3.2 Solvent Impact on Molecule Electrostatic Potential

The choice of solvent can influence the behavior of an analyte molecule in several ways that are relevant to SERS. This includes effects from the solvent on the polarity, dipole moment, and electron density distribution of the analyte molecule, all of which are related to the Raman scattering properties of the molecule. The change in the electron density distribution of the analyte molecules can then further affect the affinity of the molecules to the metallic SERS substrate.^51,52^ A solvent-induced change in the electron distribution and electrostatic potential (ESP) of the molecule implies a change in the polarizability of the molecule and, by extension, the Raman scattering properties of the molecule.^53^ Furthermore, a high-amplitude negative electrostatic potential entails a high concentration of electrons and therefore a stronger affinity for metal surfaces.^54^ For the purpose of SERS, this implies an average increase in the SERS enhancement of the molecular signals. With this in mind, we used DFT to evaluate the impact of solvation on the ESP of the nerve agent molecules.

Having calculated the ESP of VX, GA, and GF in our five different solvents using DFT, we found several commonalities in the electron distribution behavior between the three molecules. For each combination of the three nerve agents and the five solvents, the lowest ESP (and thus the highest electron density) could be found around the double-bonded oxygen atom of the P=O group (Figure 2A–C). Consequently, we expect this group to have the strongest affinity for the Au surface layer of the SERS substrate. In addition to the P=O group, our calculations predicted that each nerve agent molecule contains additional groups with notable ESP minima. The most significant example of this is GA, where the C≡N group exhibited an ESP magnitude 78.2% of the P=O group (Figure 2B) when considering water solvation. In terms of binding energy, this results in a binding energy of 0.51 eV between the P=O group and a simple gold structure, compared to 0.46 eV for the C≡N group. This suggests that between the P=O group and the C≡N group, there are two probable binding modes between GA and the metallic SERS substrate, with the P=O group being the somewhat more probable anchor site. The similarity in binding energy between these two groups could also explain the wavenumber upshifts observed in Figure 1E–G as the preferential binding mode between the GA and gold substrate may change as the surface layer is saturated, thus giving rise to two related and adjacent peaks appearing as a “shift” in the Raman spectrum. Similarly for GF, we calculated a localized ESP minimum in the P–F group (Figure 2C). The calculated ESP magnitude of this group was only 38.2% of the P=O group, and as such, the P=O group remains the much more probable binding mode. Lastly, looking at VX, we calculated an ESP minimum around the P–S–C group (Figure 2A). Similarly to GF, the ESP magnitude of the P–S–C group was much lower than that of the P=O group at only 31.5% of the P=O group, implying limited affinity for the Au SERS substrate. As such, we expect low binding energies for these side groups, and the P=O bond is expected to be the dominant binding group for both VX and GF.

**Figure 2:**
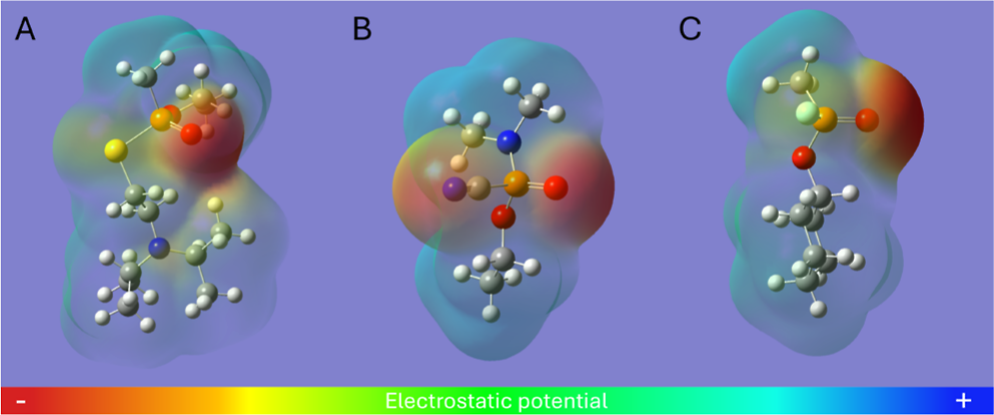
Electrostatic potential (ESP) surfaces of VX (A), GA (B), and GF (C) calculated using DFT with implicit solvation in H_2_O. Regions of the molecular geometry with negative ESP are shaded in red, scaling to blue for regions with positive ESP. The minimum ESP value for each molecule can be found in Table 2. Color scales describing ESP vary between panels A, B, and C and should not be directly compared between molecules. Atoms are depicted using typical CPK notation: gray—carbon, white—hydrogen, red—oxygen, blue—nitrogen, yellow—sulfur, orange—phosphorus, and light green—fluorine.

Looking at the differences in ESP within the same nerve agent molecule using different solvation settings, we found that the ESP magnitudes of the P=O groups were largely comparable between H_2_O, EtOH, and MeCN in all three nerve agents. In VX, we calculated only a 1.3% difference in ESP magnitude between H_2_O and EtOH. This is noteworthy as H_2_O is a significantly more polar molecule than both EtOH and MeCN, with a dielectric constant that is approximately double that of MeCN and almost quadruple that of EtOH.^55^ Looking at solvation in DCM and *n*-hexane, we found lower ESP magnitudes around the P=O group. We calculated a 3.3% decrease in the ESP magnitude of VX dissolved in DCM compared to H_2_O and a much larger 24.8% decrease between H_2_O and *n*-hexane. In fact, the calculated ESP of the nerve agents dissolved in the nonpolar *n*-hexane solvent was more similar to those calculated without a solvation model (essentially corresponding to single molecules in the gas phase), implying a much more limited interaction between the analyte and solvent. This suggests that *n*-hexane and other nonpolar solvents are likely to generate ESP magnitudes lower than those of polar solvents in similar dissolved molecules and thus lower electron concentrations in key molecular groups. For the purposes of SERS, this leads to a lower affinity between an analyte in nonpolar solvents and a metallic SERS substrate, at least for the group of molecules studied herein. This might partially explain the limited SERS activity measured in the previous section by using *n*-hexane as a solvent. A summary of the calculated ESP values of the different combinations of nerve agents and solvents is given in Table 2.

**Table 2:** Minimum ESP (×10^–2^ a.u.) of the P=O Group of Nerve Agent Molecules VX, GA, and GF, Calculated with DFT Using Different Solvation Settings

Solvent/Nerve agent	VX	GA	GF
H_2_O	–7.25	–6.45	–7.14
EtOH	–7.16	–6.40	–7.09
MeCN	–7.20	–6.42	–7.11
DCM	–7.01	–6.26	–6.96
*n*-Hexane	–5.81	–5.72	–6.39
No solvent	–5.42	–5.21	–5.96

Overall, the interplay between an analyte and a solvent plays a significant role in the SERS response of a molecule. By changing the effective ESP of certain molecular groups in the analyte, the solvents impact the vibrational properties and metallic affinity of the analyte molecule. The DFT calculations of the analyte–solvent interaction performed here illustrate this as they partially explain the experimental SERS results presented in Figure 1. The H_2_O, EtOH, and MeCN solvation models that yielded the most negative ESP values and thus the highest electron densities were indeed also those that yielded the highest SERS signal in our experiments. However, it is unlikely that this factor is solely responsible for the differences in the SERS signal observed between the different solvents. The relatively small differences in ESP magnitude between solvents (∼1%) cannot account for the large differences in SERS intensity observed between, e.g., VX in H_2_O and MeCN seen in Figure 1A,C. This may in part be due to direct chemical interactions that are not accurately described using the implicit solvation model used herein. In one example of this, the relatively hydrophobic nature of nerve agent molecules may serve to push them toward the solvent–air and solvent–substrate interfaces. In another example, the presence of protic solvents such as H_2_O or EtOH may better facilitate the formation of hydrogen bonds between the analyte molecule and the gold-coated SERS substrate compared to aprotic solvents such as MeCN or DCM. Such hydrogen bond formation would likely yield higher affinities between the analyte and the substrate, resulting in higher SERS enhancement. However, the exact influence of hydrogen bonds on molecular interactions with pristine noble metal surfaces has, to our knowledge, seen limited research so far,^56^ and it is not unreasonable to expect molecules with a lower affinity for gold to even be out-competed by the solvent for the limited number of binding sites on the substrate.^19^ Furthermore, given another set of solvents and SERS substrates, the SERS signal using MeCN has been shown to be as good as that using EtOH, as previously demonstrated by Tripathi et al.,^19^ if not better. Therefore, while it is important to consider the interactions between the analyte molecule and solvent (for example, using DFT models as done herein) when choosing a solvent for SERS measurements, it is equally important to consider potential interactions between the solvent and the SERS substrate.

### 3.3 Solvent Effects on Analyte Distribution and Nanopillar Geometry

In designing a SERS detection assay, several factors need to be considered when choosing an appropriate substrate for the assay. The choice of plasmonic material is a good example of this; while pristine silver is often an excellent choice for a strong SERS enhancement, it is also susceptible to carbon contamination, sulfidation, and oxidation issues, the latter especially in the presence of more acidic environments and solvents.^57–59^ Similarly, interactions between solvent and SERS surface nanogeometry may make certain solvents more appropriate for use with a given geometry than others. This interaction can take two forms. First, there is the action from the surface on the droplet, where the surface energy will determine the shape and dynamics of the droplet and in turn the surface concentration of the analyte. Second, there is the action of the droplet on the surface, where the capillary forces from the droplet evaporation process affect the morphology of the SERS substrate. In our experiments, we used relatively hydrophobic SERS surfaces printed with semielastic silicon nanopillars and covered in a layer of Au. These nanopillars have previously been shown to aggregate when exposed to the capillary forces of an evaporating sample droplet, forming SERS hotspots.^22,43^ Considering what we know about these substrates, it is thus worth investigating whether and how the choice of solvent impacts the surface concentration of the analyte and SERS hotspot formation due to nanopillar aggregation related to forces caused by the droplet evaporation.

To measure how the choice of solvent affects the droplet properties of the sample deposition process, we used an optical tensiometer to evaluate the contact angle between the solvent droplet and the surface. We found that the choice of solvent had a significant impact on the contact angle, with H_2_O displaying by far the highest average contact angle with the SERS surface at 126°. In practice, this corresponds to a largely spherical droplet covering a limited section of the SERS substrate, as shown in Figure S17. This droplet shape causes a larger number of analyte molecules to be concentrated in a smaller area in the middle of the SERS substrate, resulting in a higher analyte surface concentration. From a SERS perspective, this is likely to result in higher SERS intensities as more molecules are available on the surface. However, it may also result in the formation of multilayers at higher analyte concentrations, resulting in a loss of signal, as seen in Figure 1D. In comparison, the other solvents formed much lower contact angles with the surface, with EtOH and *n*-hexane both averaging 23°, while MeCN and DCM formed even lower contact angles, averaging 17° and 12°, respectively. Furthermore, since these droplets in several cases were in contact with the edge of the SERS surface, it is not unthinkable that even lower contact angles could have formed on SERS surfaces with a larger surface area. These lower contact angles resulted in much more horizontal droplets that cover the entire surface of the SERS substrate, as demonstrated in Figure S18. Consequently, we expect these lower contact angles to yield a more homogeneous surface concentration of the analyte over the substrate.

Another factor that should be considered is the time and mechanism through which the solvents evaporate from the surface. All of the used solvents exhibited some degree of stick–slip drying on the nanopillar substrate, which means that the contact line between the droplet and the surface receded not strictly continuously, but rather in discrete steps.^60^ This phenomenon was especially noticeable in MeCN, where the evaporation often did not result in a visible inward recession, but rather a simultaneous evaporation of the remaining MeCN once a sufficiently low contact angle is produced. The stick–slip effect also had a visible effect on the H_2_O sample, where it led to the formation of noticeable “coffee rings” on the substrate, inferring larger local concentrations of analyte.^21^ In comparison, EtOH generally displayed few or a single “slip”, resulting in a delayed, albeit mostly continuous, recession of the droplet. The time it takes for the droplet to evaporate will naturally also influence the degree to which the analyte can equilibrate on the substrate, as well as the direct action of the solvent on the surface. Measurements of the evaporation time of the solvents on the substrates showed that H_2_O had the longest evaporation time by a large margin at 1048 s (∼17.5 min). For comparison, we recorded evaporation times of 64 and 60 s for EtOH and MeCN, respectively, and only 16 and 10 s for DCM and *n*-hexane, respectively. The longer evaporation time of H_2_O is likely beneficial for SERS as it gives the analyte molecules more time to equilibrate and evenly distribute across the uneven topography of the SERS substrate, especially at low analyte concentrations. In practice, we would therefore expect a relatively even analyte distribution across the parts of the substrate covered by the H_2_O droplet, with marginally more analyte around the center of the surface as the area around the center will hold the crest of the droplet and thus more analyte projected above it. In contrast to H_2_O, the faster evaporation of the other solvents allows the analyte less time to equilibrate onto the surface and into any SERS hotspots, resulting in a lower SERS signal. This factor likely contributed to the low Raman activities measured using DCM and *n*-hexane as solvents. Finally, EtOH and MeCN, which evaporated in around a minute, yielded intermediate SERS signal intensities compared to those of H_2_O and DCM/*n*-hexane (as previously presented in Figure 1). The contact angles and evaporation times discussed are summarized in Table 3.

**Table 3:** Average Contact Angles and Evaporation Times of Different Solvents on Au Nanopillar SERS Substrates, alongside Standard Deviations (SD)

Solvent	Contact angle		Evaporation time	
	Angle (deg)	SD	Time (s)	SD
H_2_O	126	5	1048	43
EtOH	23	6	64	6
MeCN	17	2	60	2
DCM	12	2	10	1
*n*-hexane	23	6	16	2

Moving on to the action from the solvent onto the substrate, we consider the tangential force applied from the solvent onto the nanopillars. It is this tangential force that causes the nanopillars to bend into each other and aggregate, forming SERS hotspots. In a previous work by Das et al. it was demonstrated that the capillary forces of a droplet acting on a surface include an inward tangential component, acting from the solvent–surface–air interface toward the center of the droplet.^61^ This tangential force component scales with the contact angle between the liquid and the surface, reaching its maximum at contact angles >90^°^. Therefore, a higher contact angle between the solvent and the SERS substrate should result in a larger tangential force on the nanopillar geometry, causing the nanopillars to bend into clusters and form SERS hotspots. Based on our previous observations on the contact angles between our SERS substrate and the different solvents, we can infer that H_2_O should project the largest action from the droplet on the nanopillar structure. The much larger contact angles between H_2_O and the SERS substrate compared to the other solvents, together with the long evaporation time, should inflict the most action on the nanopillars and thus cause the most prominent clustering of nanopillars. In comparison, EtOH and MeCN should have less impact on the nanomorphology of the SERS substrates, while the short evaporation time of DCM and *n*-hexane will even more severely limit the impact of the solvents on the substrate. Because of this, we would expect to see the largest formation of SERS hotspots from samples dissolved in H_2_O, followed by EtOH and MeCN, and limited to no formation from DCM and *n*-hexane.

To evaluate the impact of the solvents on the nanomorphology of the substrates, we performed SEM imaging before and after deposition and evaporation of the different solvents. Looking at pristine SERS substrates, we found largely independent nanopillars and very few nanopillar clusters that could act as SERS hotspots, as seen in Figure 3A. Upon deposition of solvent on the top of the substrates, we found notable changes in the nanomorphology of the substrates. Especially H_2_O led to significant aggregation of the nanopillars (Figure 3B). Specifically, Figure 3B shows the nanomorphology of the nanopillar structure close to the edge of the deposited droplet. Here, we can tell a clear difference between the area affected by the capillary forces from the solvent (top of the image) and the area unaffected by the solvent (bottom of the image), where the part of the substrate affected by the capillary forces from the solvent has seen significant deformation and nanopillar aggregation. These nanopillar aggregations are largely continuous over the solvent-exposed parts of the substrate, with shorter distances between each pillar and its nearest neighbors compared to those of the pristine substrate. This likely plays a large role in the high SERS intensities observed in Figure 1 when using H_2_O as a solvent and is in line with the previous work by Das et al.^61^ Some nanopillar aggregation was also found after the deposition of EtOH (Figure 3C) and to a lesser extent MeCN (Figure 3D). These aggregations were limited to individual clusters, rather than the continuous aggregation observed throughout the H_2_O-covered substrate. In terms of frequency, we estimate ∼3–4 clusters/μ m^2^ of 3 or more nanopillars after deposition of EtOH. This number was noticeably lower after deposition of MeCN, at only ∼1 cluster/μ m^2^. This is in line with our results in Figure 1D,H,L, where EtOH consistently shows SERS intensities 2–5 times higher than MeCN when used as a solvent. However, one should be prudent not to draw too large parallels between the number of hotspots and the resulting SERS intensity, given that small changes in the distribution of nanostructures can have a large impact on SERS enhancement and, while the number of discrete clusters is a factor in this enhancement, it does not by itself quantify the level of enhancement. Moving on to DCM and *n*-hexane (Figure 3E,F), we found little or no impact of the solvent on the nanomorphology of the substrate. This is in line with our previous measurements on contact angle and evaporation time; as both solvents showed very low contact angles with the substrate (∼12–23°) and a very short evaporation time (10–15 s), there is very little force acting from the solvents on the nanopillars. The lack of clusters is also consistent with our SERS measurements as the lack of clusters minimizes the number of SERS hotspots and therefore SERS activity. This further confirms that DCM and *n*-hexane are less suitable solvents for similar SERS applications on nanopillar geometries. Although these recorded morphological effects are specific to this particular nanogeometry, one should be alert to similar morphological effects when observing large contact angles on other SERS substrates. Overall, because of the impact of the solvent on the analyte surface concentration and SERS substrate morphology, the SERS profile over the substrate is expected to vary greatly between solvents.

**Figure 3:**
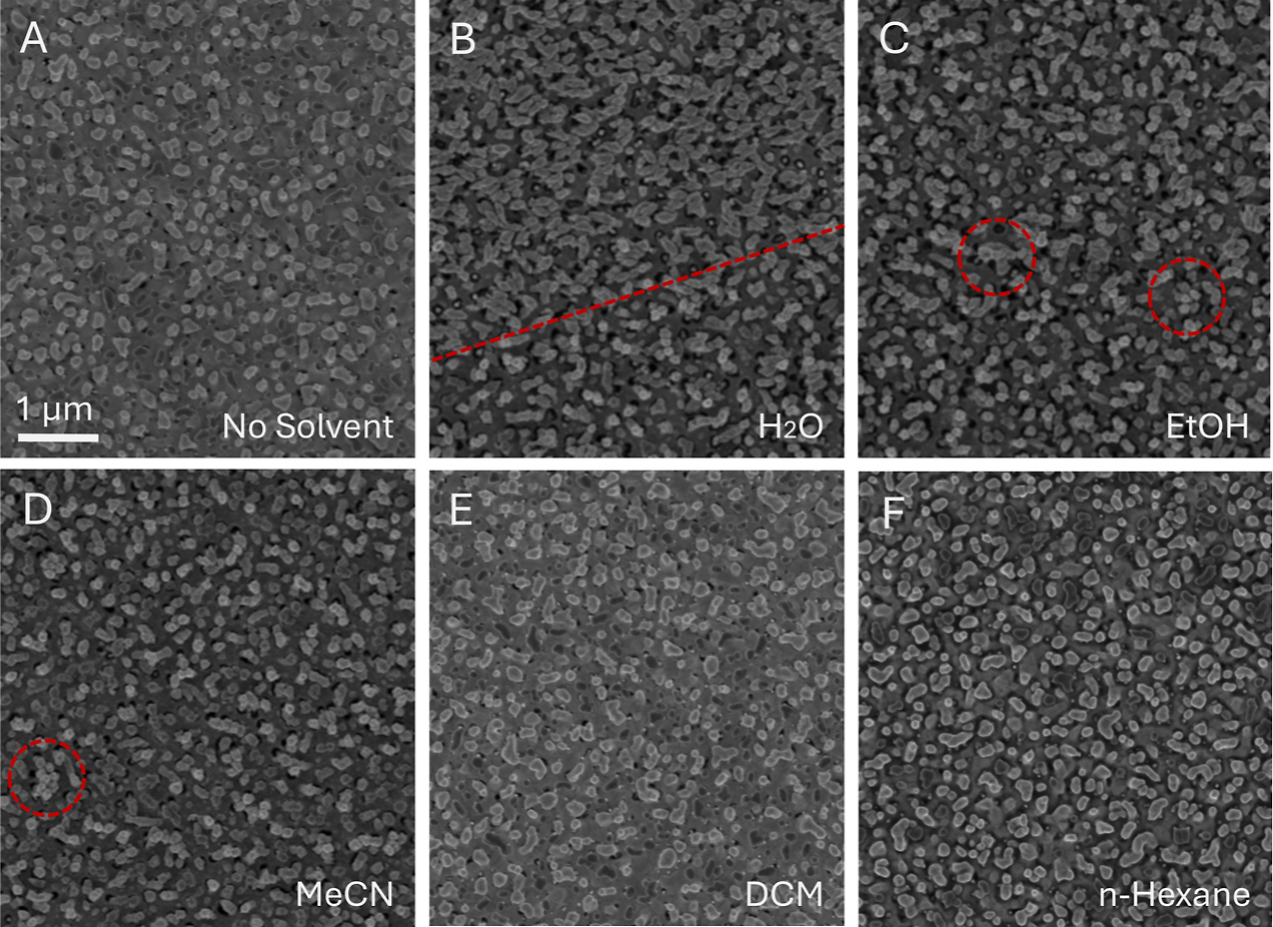
SEM images of the Au-covered nanopillar SERS substrate before (A) and after deposition of H_2_O (B), EtOH (C), MeCN (D), DCM (E), and *n*-hexane (F). The red dashed line in B denotes the approximate border between the aggregated and nonaggregated nanopillars on the SERS substrate. Red circles in (C) and (D) highlight the local nanopillar clusters.

To explore the effects of the different solvents on the SERS signal distribution on the nanopillar substrate from different solvents, we used Raman mapping. As no SERS signal was obtained from any of the nerve agents studied using DCM or *n*-hexane as solvents, we limited these measurements to only H_2_O, EtOH, and MeCN. Mapping the VX 900 cm^–1^ band from 3 ppm VX dissolved in H_2_O, we found that the SERS-active area of the SERS substrate naturally corresponded to the area of the SERS substrate covered by the H_2_O droplet (Figure 4A). There were two parts of the SERS substrate with a notably enhanced SERS signal compared to the rest of the substrate; the center of the deposited droplet and around the border of the droplet. The increased SERS signal in the center of the droplet is intuitive as the droplet dries inward toward the center of the droplet, resulting in a higher surface concentration of the analyte around the center of the dried droplet. This effect has previously been demonstrated by, e.g., Hakonen et al.^43^ Meanwhile, the increased activity around the edge of the droplet could be caused by the high tangential forces experienced by the nanopillars along the initial solvent–air interface, producing more hotspots and resulting in an increase in the SERS signal compared to the parts of the surface closer to the center of the droplet.^61^ There was also a slight coffee ring effect that could be visually observed on the substrate, likely caused by the combination of long evaporation times and the stick–slip evaporation mechanic, suggesting possible inhomogeneities in the analyte distribution along the edge of the droplet. These inhomogeneities in the distribution of the SERS signal likely account for the large signal variance observed when using H_2_O as a solvent in Figure 1D,H,L. Looking at other solvents for comparison, the VX 900 cm^–1^ signal was present throughout the whole SERS substrate when EtOH and MeCN were used as solvents. We hypothesize that the lower contact angles of these solvents allow for a more even surface concentration of the analyte on the SERS surface. Notably, the intensity of the SERS signal was higher toward the center of the SERS surface in the EtOH-dissolved sample, compared to the MeCN-dissolved analyte where a more intense SERS signal was acquired around the edge of the SERS surface. This likely stems in part from differences in the way the two different solvents evaporate on the substrate, with the higher-polarity EtOH more likely to dry inward as a coherent droplet in a single “slip” within the final seconds of evaporation, whereas MeCN evaporates over the substrate largely simultaneously with less noticeable droplet recession. Any remnants of the analyte on the edge of the SERS surface may then be more heavily affected by edge effects, plausibly resulting in stronger SERS enhancement. It should be noted that this evaporation dynamic may play out differently on other SERS substrates with different surface energies and should thus be re-evaluated whenever designing a new SERS assay. Similar trends could also be observed from the mapping characteristic 2150 and 760 cm^–1^ bands of GA and GF, respectively (Figure S19). All of these factors contribute to a significant variance in signal intensity over the span of the SERS substrate. Specifically, using EtOH and MeCN as solvents, we found a relatively low variance in the SERS signal, with relative standard deviations of 20.4 and 20.5%, respectively. The signal variance when using H_2_O was significantly greater; with a relative standard deviation of 28.1% when considering only points within the established “droplet area” in Figure 4A and a massive 138.2% when considering the entire substrate. The large deviation in the latter case is, of course, a function of including points where no signal is expected. Overall, these results show that the choice of solvent and the way it interacts with the SERS substrate have a significant impact on the intensity and distribution of the SERS signal. They illustrate that when designing a SERS measurement procedure, it is important not only to choose the right solvent for a specific measurement but also to design the measurement procedure around the chosen solvent; e.g., by adapting data processing algorithms and procedures to the inherent distribution of the SERS signal characteristic of the given solvent. This becomes even more important with the continued development of SERS mapping tools, more powerful data processing algorithms, and a growing need for practical adaptability to bring SERS measurements to the field.

**Figure 4:**
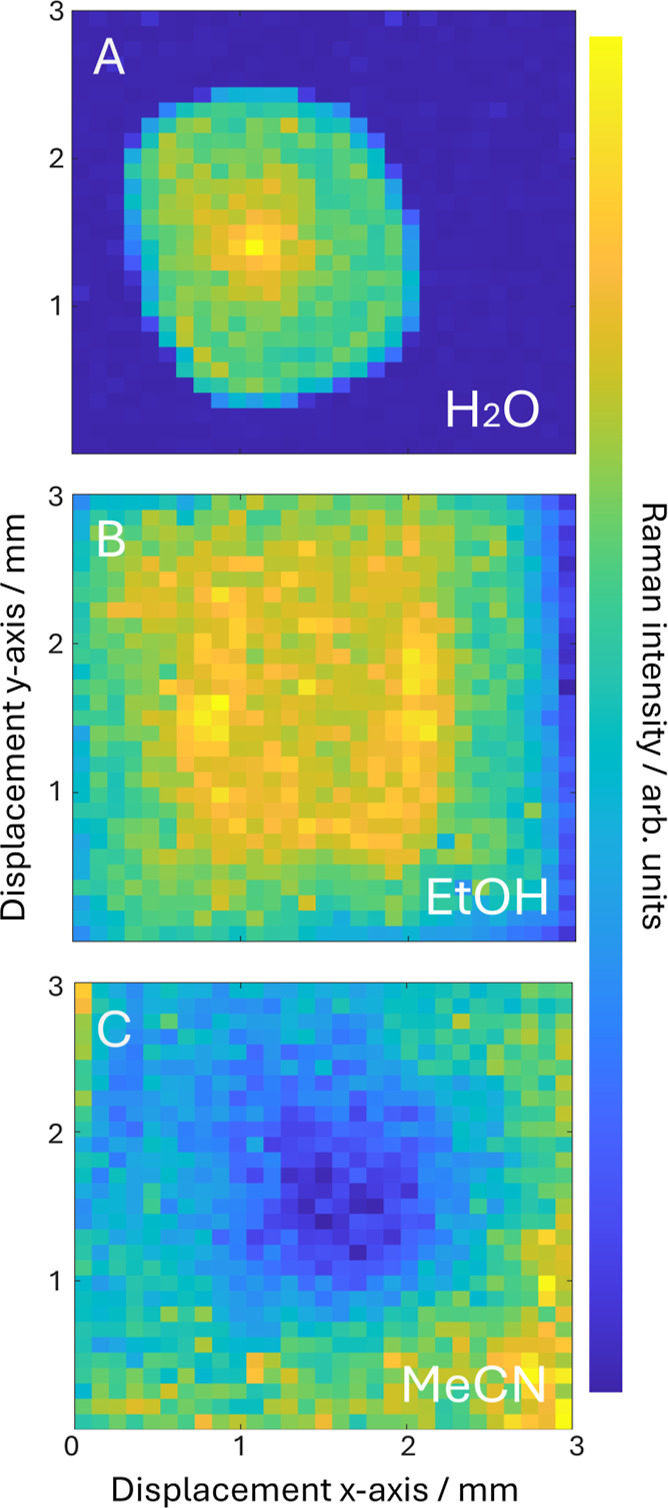
Raman maps of 3 ppm VX on Au-coated nanopillar SERS substrates using H_2_O (A), EtOH (B), and MeCN (C) as solvents. The absolute Raman scattering intensity varied significantly between the solvents, as illustrated in Figure 1D. Yellow pixels indicate high SERS activity, while blue pixels indicate low activity.

### 3.4 Additional Concerns for Practical SERS Detection Purposes

As presented in this work, the choice of solvent is an important parameter in optimizing an SERS detection assay. For example, the polarity of the chosen solvent will impact the effective electrostatic potential of the analyte, as illustrated herein using DFT, and thus its vibrational properties and interactions with other materials. In the same vein, we have shown that H_2_O generates by far the most force from the solvent on the SERS substrate, facilitating the formation of SERS hotspots in similar SERS geometries. Although these factors alone may lead one to assume that H_2_O is the generally optimal choice in SERS detection scenarios, it is important to consider other practical implications that the choice of solvent will have on a detection assay. One of these implications is the effect of variance. As demonstrated in Figure 1D,H,L, we generally recorded a higher variance when using H_2_O as a solvent, which is further supported by the significant spatial spread in intensity observed in Figure 4A. This larger variance may prove problematic when, e.g., algorithms or models are constructed to recognize and match Raman spectra to known references. Another relatively simple consideration is that of solubility as good analyte solubility is required to effectively deposit the analyte on the SERS substrate. This consideration is key in the context of this work as H_2_O is a particularly polar solvent and thus not appropriate as a solvent for a range of nonpolar molecules. Also important to consider is whether analyte pretreatment (e.g., filtering or reconcentration) is necessary ahead of SERS measurement. Methods such as liquid–liquid extraction can be highly useful in sample pretreatment but often require transferring the sample through one or several additional solvents, complicating the solvent and analyte properties of the sample as they relate to SERS.^62^

Another practical consideration is the time required to measure a nerve agent sample. One of the main benefits of SERS is that it can provide a fast and specific detection of a given analyte. However, less volatile solvents, such as H_2_O, naturally take longer to dry on a SERS surface, as shown in Table 3. Although the additional 10–15 min of evaporation time for a droplet of H_2_O under ambient conditions would likely not be critical in some SERS applications, applications that require extremely fast response times may find such a wait infeasible. The detection of nerve agents documented in this work is an example of this. Field applications of SERS for the detection of nerve agents, especially in areas of active conflict, must be carried out as quickly as possible to minimize the risk of exposure to the agent or surrounding hazards and facilitate the rapid treatment of exposed personnel.^63^ For this purpose, EtOH is a good compromise solvent, showing a relatively low LOD while simultaneously facilitating much faster sample identification and a lower spectral variance than H_2_O. This further illustrates the multitude of considerations required when designing a SERS assay and that although one solvent may be preferable strictly to achieve a lower LOD (as with H_2_O in our case), practical aspects may lead to other solvents being more optimal for many practical applications.

## 4 Conclusions

Low-volatility nerve agents, such as VX, GA, and GF, are highly lethal even in trace amounts. One promising method for detecting low-volatility nerve agents is SERS; however, the SERS characteristics of a sample may significantly depend on the solvent in which the analyte is dissolved. Although it is well-known that the choice of solvent will affect the SERS characteristics of an analyte, its full impact on both solvent–analyte interactions and solvent–substrate interactions is rarely given full consideration. In this work, we have investigated how the SERS characteristics of the nerve agents mentioned above are affected by different solvents, using H_2_O, EtOH, MeCN, DCM, and *n*-hexane as examples. More specifically, we investigated how variations in SERS spectral characteristics for different solvents correlate with both solvent–analyte interactions and solvent–substrate interactions between the solvent and the nanopillar-patterned SERS surface. We found that for all nerve agents, H_2_O was the most effective solvent in terms of detection limit, especially for VX, where the LOD was found to be as low as 0.2 ppm. Following H_2_O, EtOH and MeCN were found to have LODs of 8.7 and 6.3 ppm, respectively, although the latter in general displayed low signal intensities, which may prove problematic in real detection applications. DCM and *n*-hexane did not show significant spectral activity when used as solvents for any nerve agent used herein. Using DFT, we found that the high SERS intensity observed when H_2_O is used can be partially explained by its high polarity. High-polarity solvents such as H_2_O were found to yield higher electron concentrations in the reactive P=O groups of the nerve agent molecules, affecting their vibrational properties and boosting their affinity for metal surfaces such as those on the SERS substrate. This should, in turn, yield larger SERS enhancement. However, the interaction between the solvent and SERS substrate is arguably more impactful. Using contact angle measurements, we found that the droplets formed from H_2_O and, to a lesser extent, EtOH and MeCN impose a large tangential force from the droplet onto the SERS substrate. Using SEM, we demonstrated how this caused the nanostructures of the SERS substrates to bend and aggregate, forming SERS hotspots. The reason why this is particularly prominent in H_2_O is likely due to the high contact angle between the H_2_O droplet and the SERS substrate, generating a larger tangential force onto the nanopillars, causing them to aggregate into hotspots. Overall, the SERS characteristics presented herein using H_2_O as a solvent, in addition to its combination of a high polarity and a large contact angle, suggest that H_2_O is a very appropriate solvent for SERS applications, in accordance with conventional wisdom and using similar SERS geometries. However, these conclusions are no substitute for sound experimental design, and the prudent researcher should always consider the parameters of their experiments and use-related restrictions. For example, in many practical applications, relatively homogeneous SERS signal distributions and lower evaporation times, such as those observed in EtOH and MeCN, may often take precedence over the lower LOD of H_2_O. Furthermore, factors such as compatibility with relevant chemicals and evaporation time in relation to the use case limitations are crucial to consider when weighing an appropriate solvent for SERS experiments. We hope that the results presented herein may provide a useful starting point in choosing an appropriate solvent for future SERS experiments.

## Supplementary Material



## Notes

Additional and complete Raman spectra of analyte–solvent combinations used herein; fitting parameters for the curves in Figure 1D,H, L; example images from contact angle measurements; and Raman maps of GA and GF in water, ethanol, and acetonitrile (PDF)
