# Supplementary material for: Impact of Solvent Choice on SERS Sensing: Solvent–Analyte and Solvent–Substrate Effects for Organophosphate and Nerve Agent Detection
Source: J Phys Chem A. 2026 Aug 3;130(32):6343–55. doi: 10.1021/acs.jpca.6c02753 (PMC13479609; doi:10.1021/acs.jpca.6c02753)
Supplement: Supplementary file 1 [file jp6c02753_si_001.pdf]

Impact of Solvent Choice on SERS Sensing: Solvent-Analyte and Solvent-Substrate Effects for Organophosphate and Nerve Agent Detection

Rasmus Öberg[a,b], Jonas Segervald[b], Andreas Larsson[a], Magnus Andersson[b,c], Per Ola Andersson[a,*]

[a] Swedish Defence Research Agency (FOI), Umeå, 90621, Sweden
[b] Department of Physics, Umeå University, Umeå, 90736, Sweden
[c] Umeå Centre for Microbial Research (UCMR), Umeå, 90736, Sweden
* Corresponding author

Additional supporting tables 2

Additional supporting figures 3

Additional supporting tables

Table S1: Fitting parameters for concentration vs. Raman intensity plots from Figure 1.

Nerve agent	Solvent	A	k	n	R^2-value
VX	H_2O	2.196	0.705	1.07	0.915
	EtOH	2.751	8.111	1.01	0.986
	MeCN	0.783	3.498	1.57	0.900
GA	H_2O	9.416	252.745	1.55	0.943
	EtOH	Could not be accurately fitted			
	MeCN	1.063	312.384	0.861	0.926
GF	H_2O	2.232	600.577	2.26	0.979
	EtOH	1.978	858.082	1.92	0.907
	MeCN	0.701	1168.010	1.42	0.907

Additional supporting figures

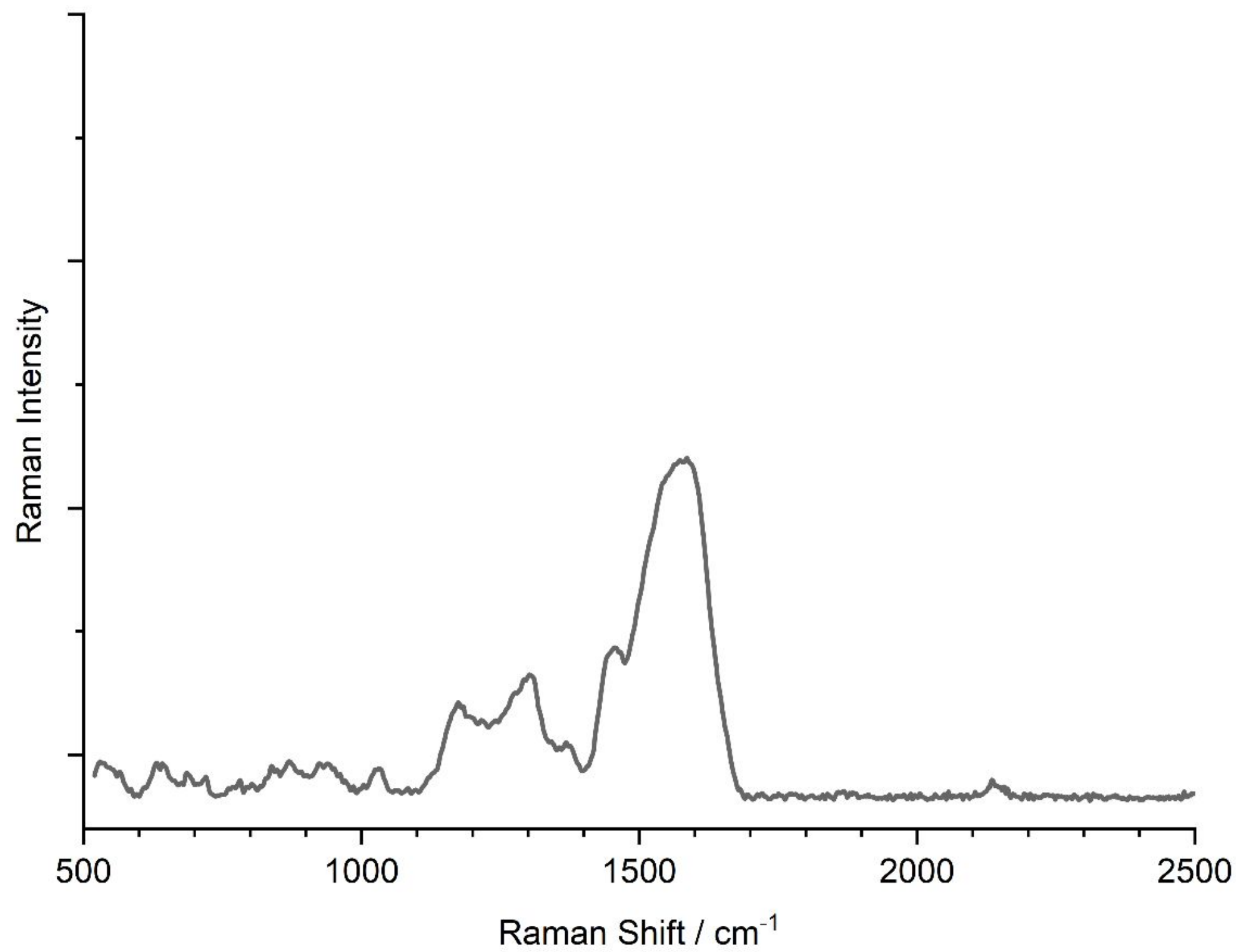

Figure S1: Averaged SERS spectrum of pristine SERS substrate.

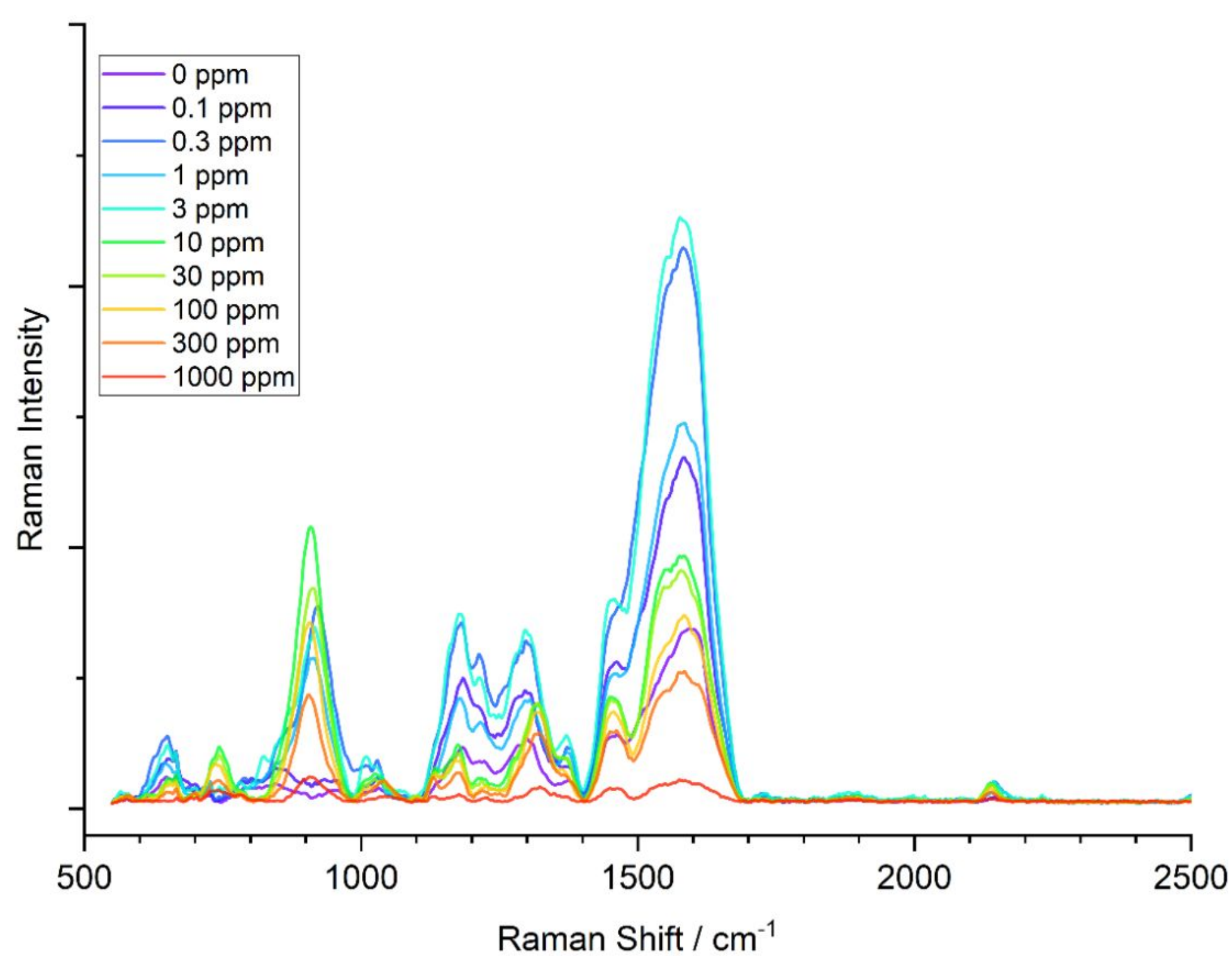

Figure S2: SERS spectra of VX dissolved in water at increasing concentrations.

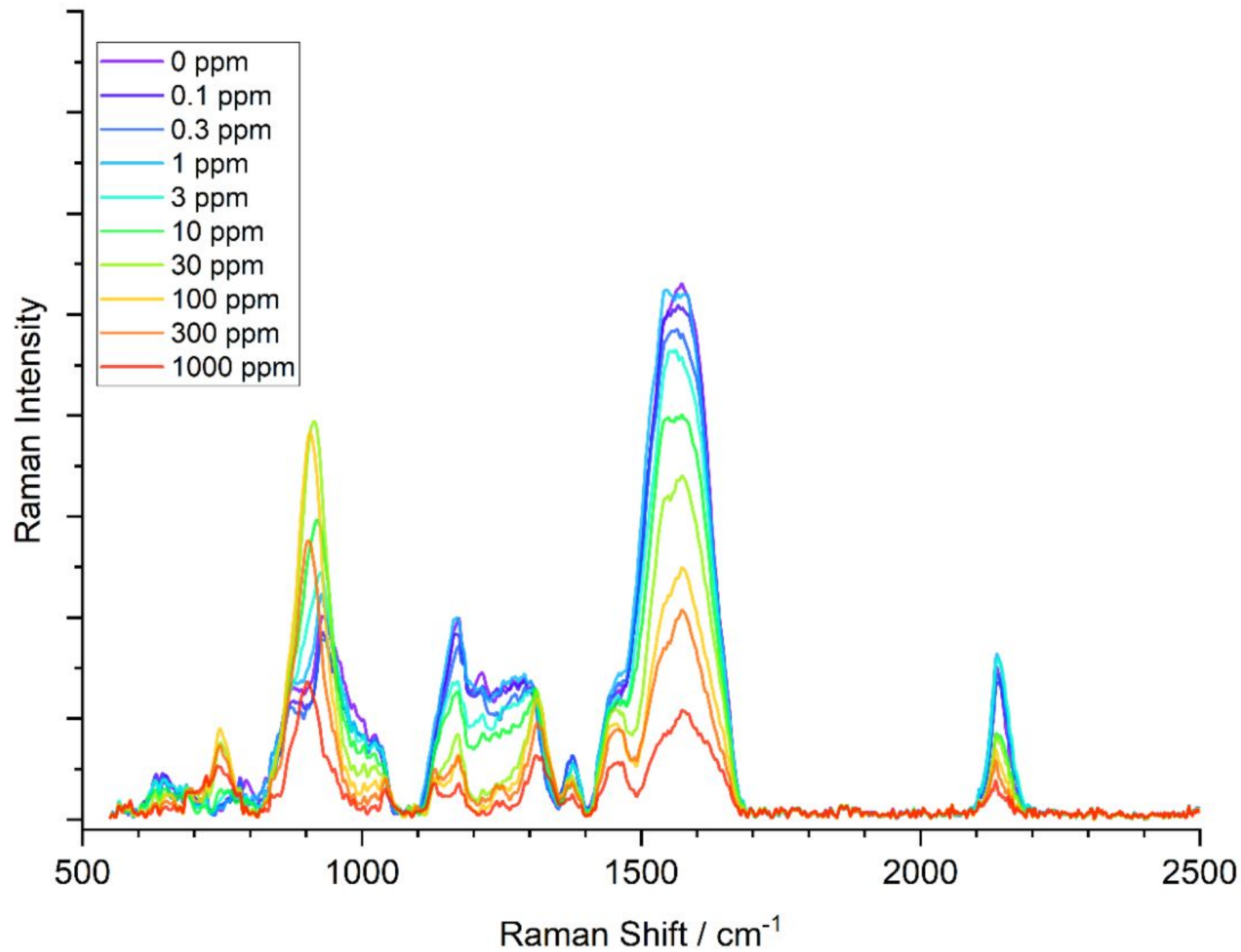

Figure S3: SERS spectra of VX dissolved in EtOH at increasing concentrations.

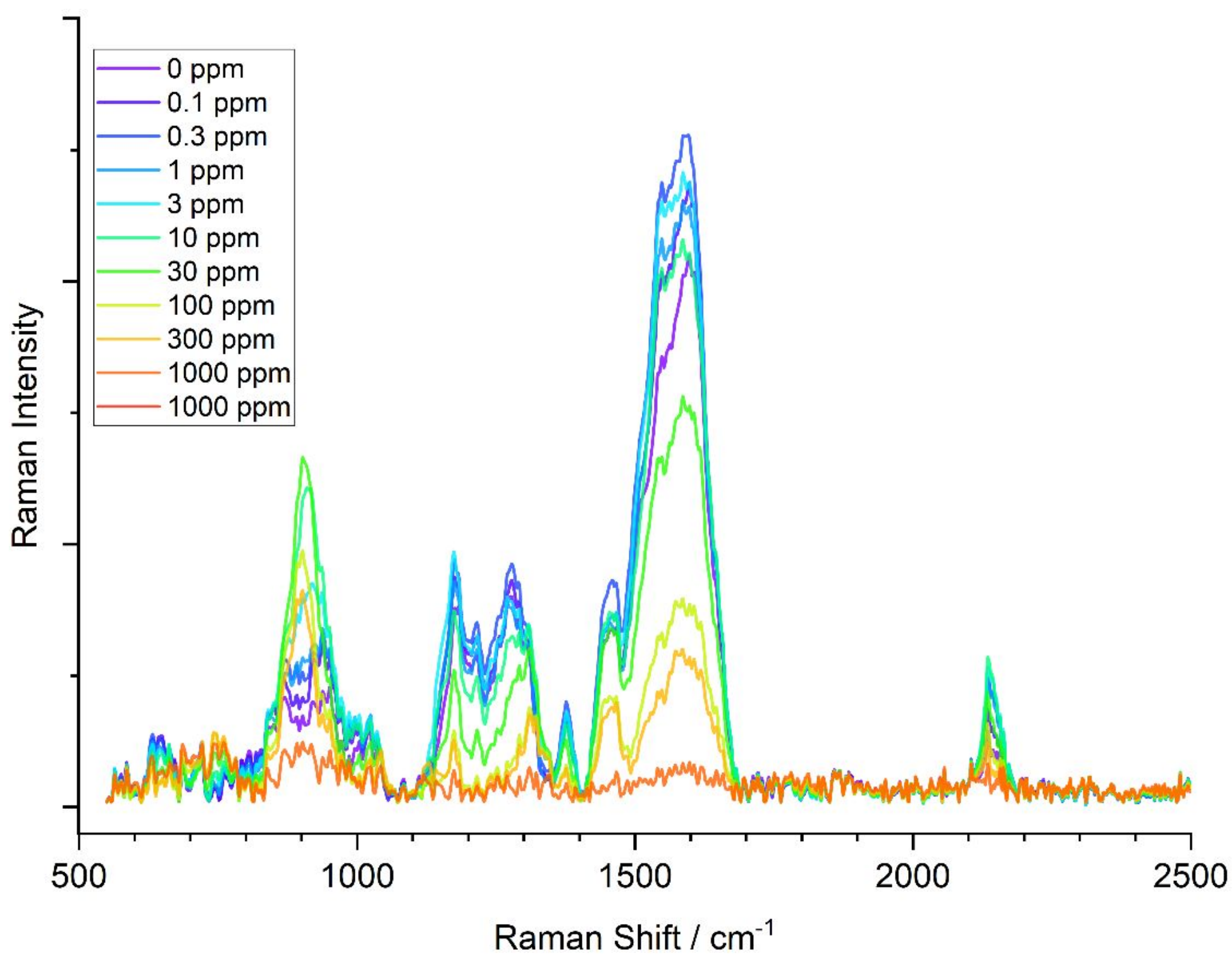

Figure S4: SERS spectra of VX dissolved in MeCN at increasing concentrations.

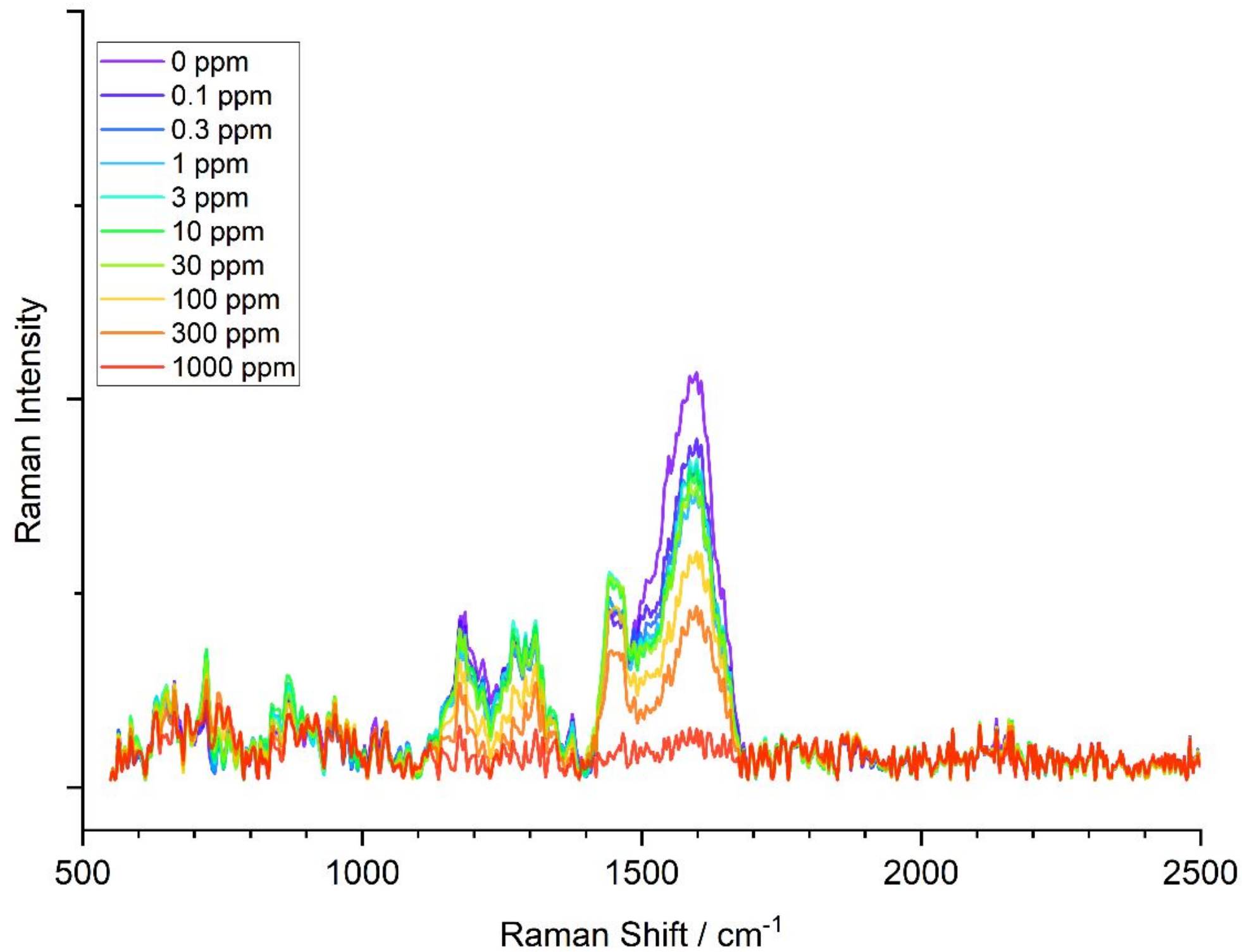

Figure S5: SERS spectra of VX dissolved in DCM at increasing concentrations.

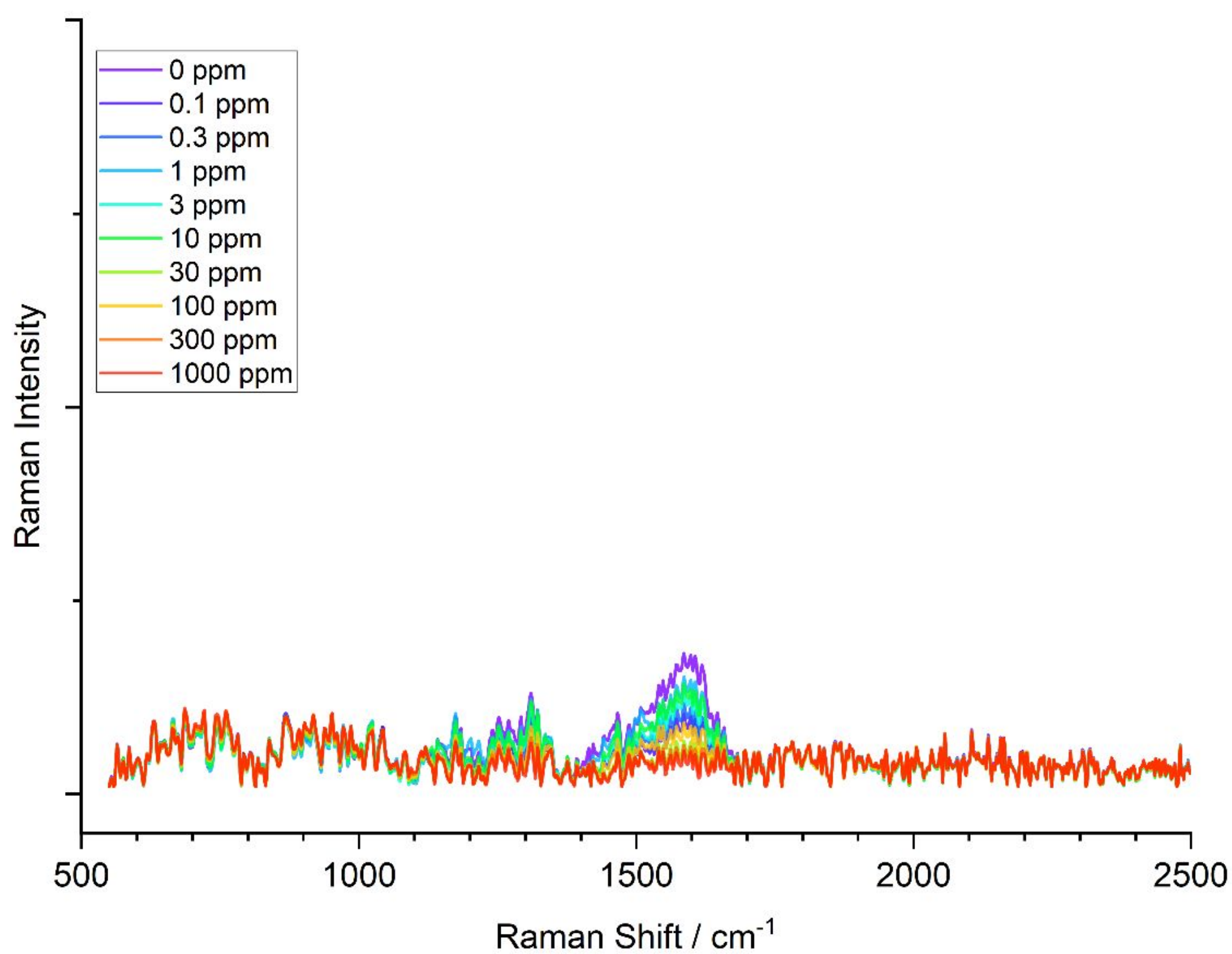

Figure S6: SERS spectra of VX dissolved in n-hexane at increasing concentrations.

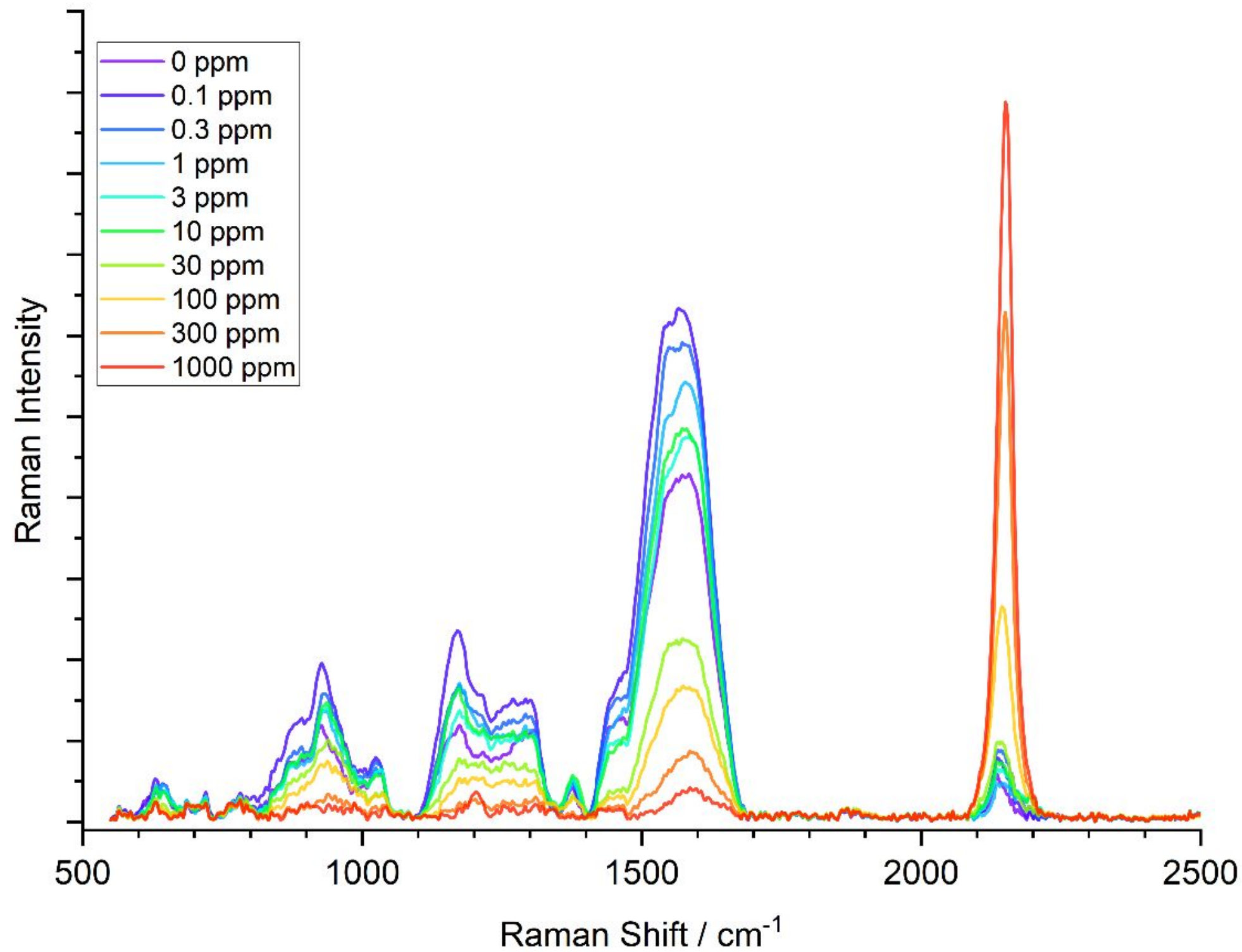

Figure S7: SERS spectra of GA dissolved in water at increasing concentrations.

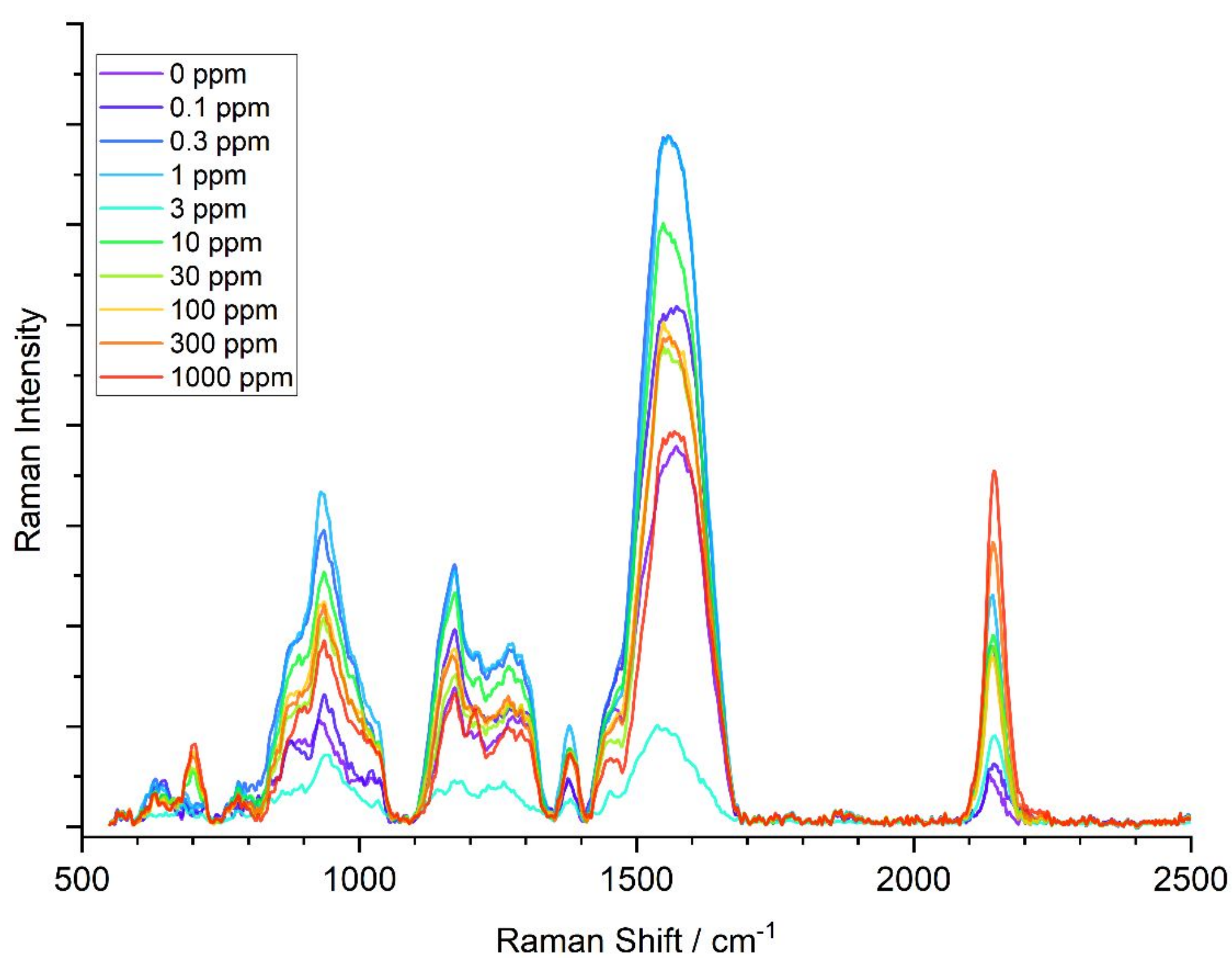

Figure S8: SERS spectra of GA dissolved in EtOH at increasing concentrations.

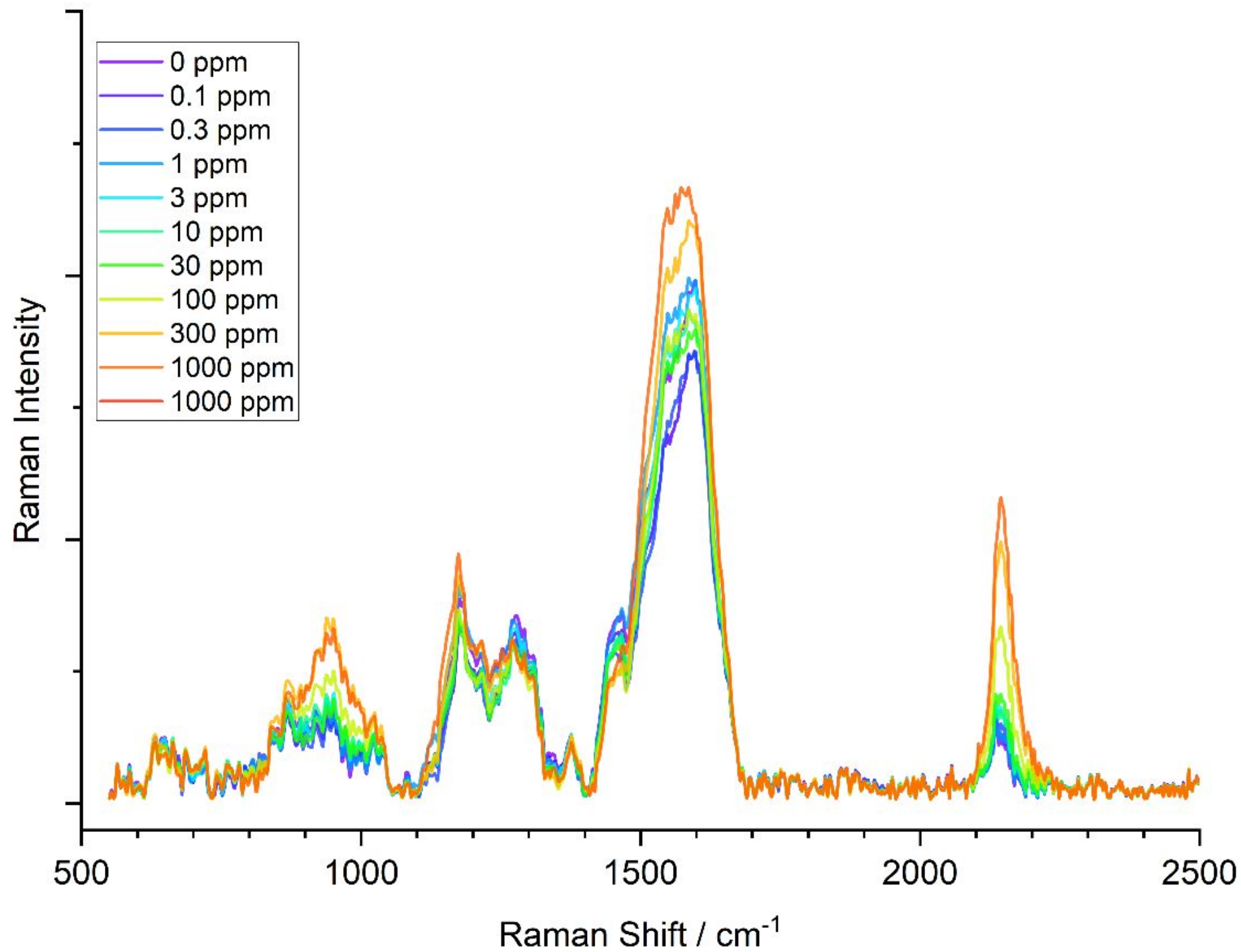

Figure S9: SERS spectra of GA dissolved in MeCN at increasing concentrations.

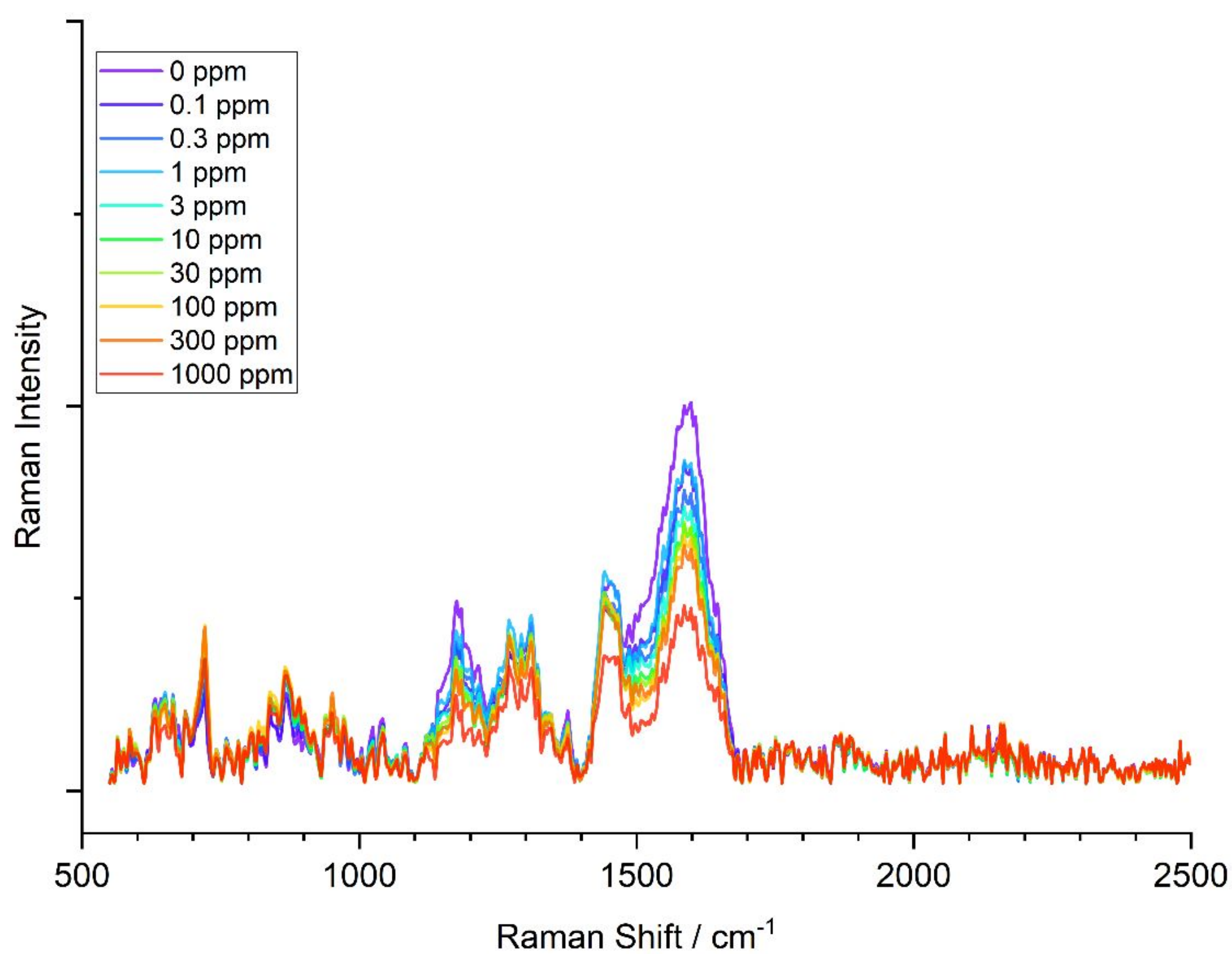

Figure S10: SERS spectra of GA dissolved in DCM at increasing concentrations.

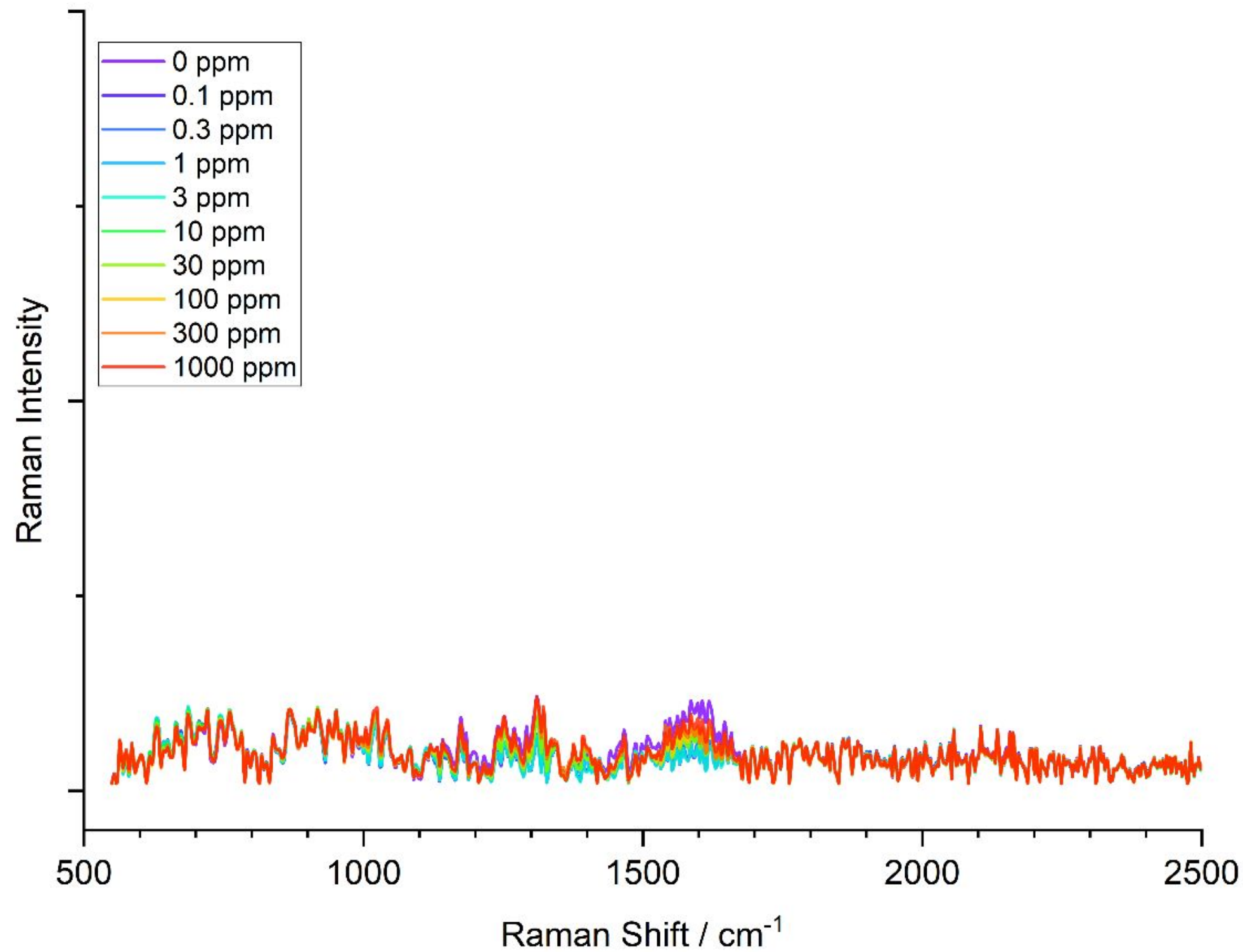

Figure S11: SERS spectra of GA dissolved in n-hexane at increasing concentrations.

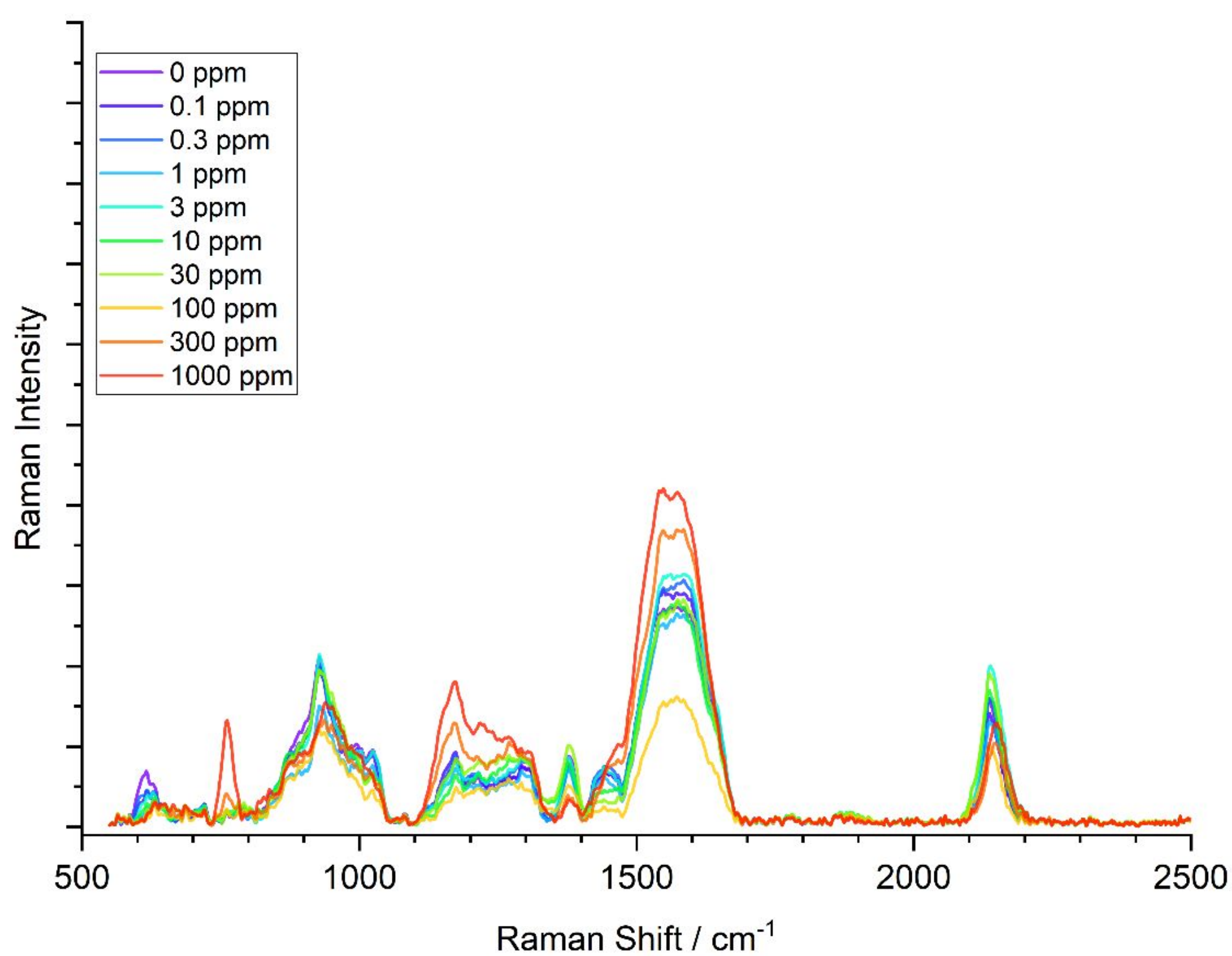

Figure S12: SERS spectra of GF dissolved in water at increasing concentrations.

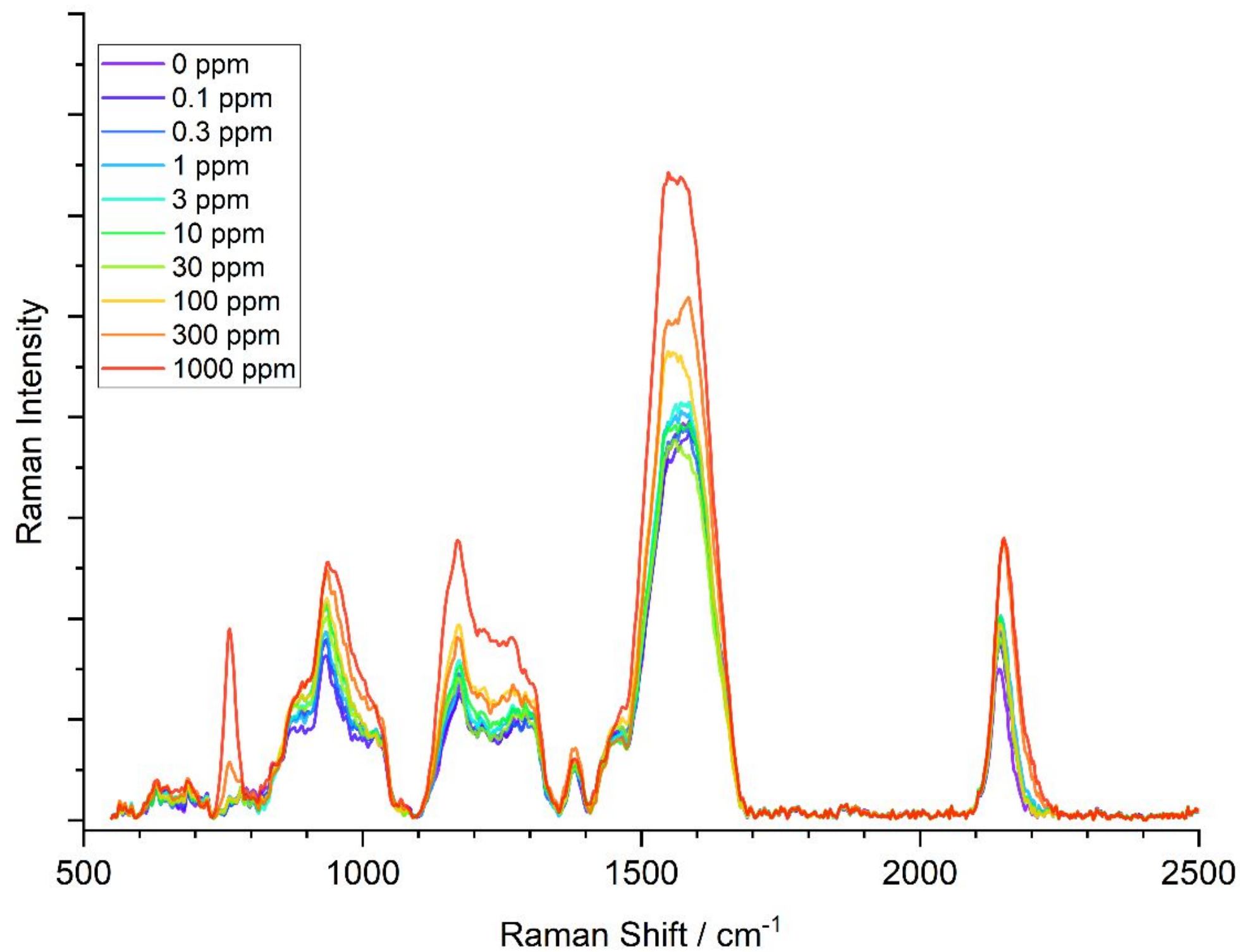

Figure S13: SERS spectra of GF dissolved in EtOH at increasing concentrations.

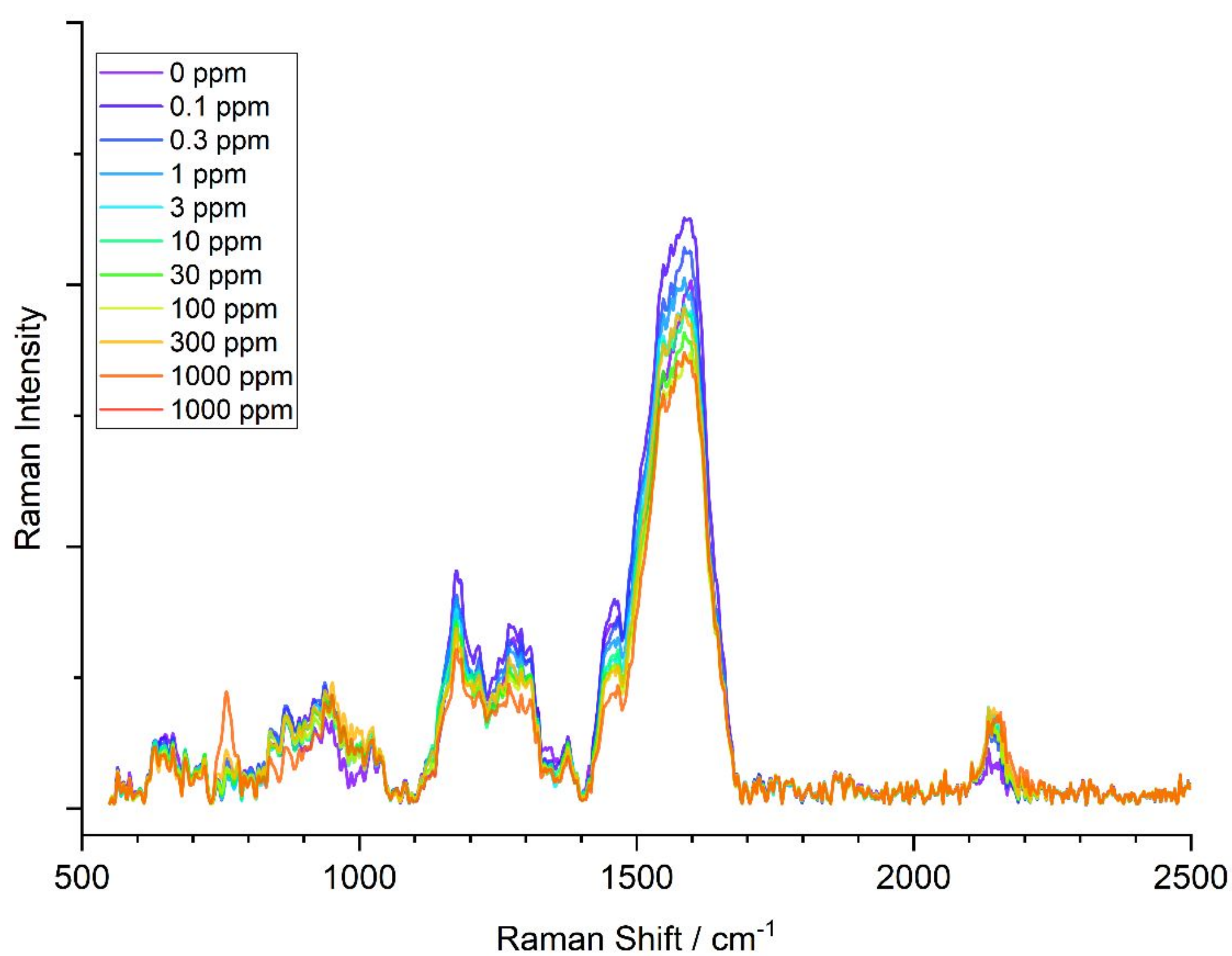

Figure S14: SERS spectra of GF dissolved in MeCN at increasing concentrations.

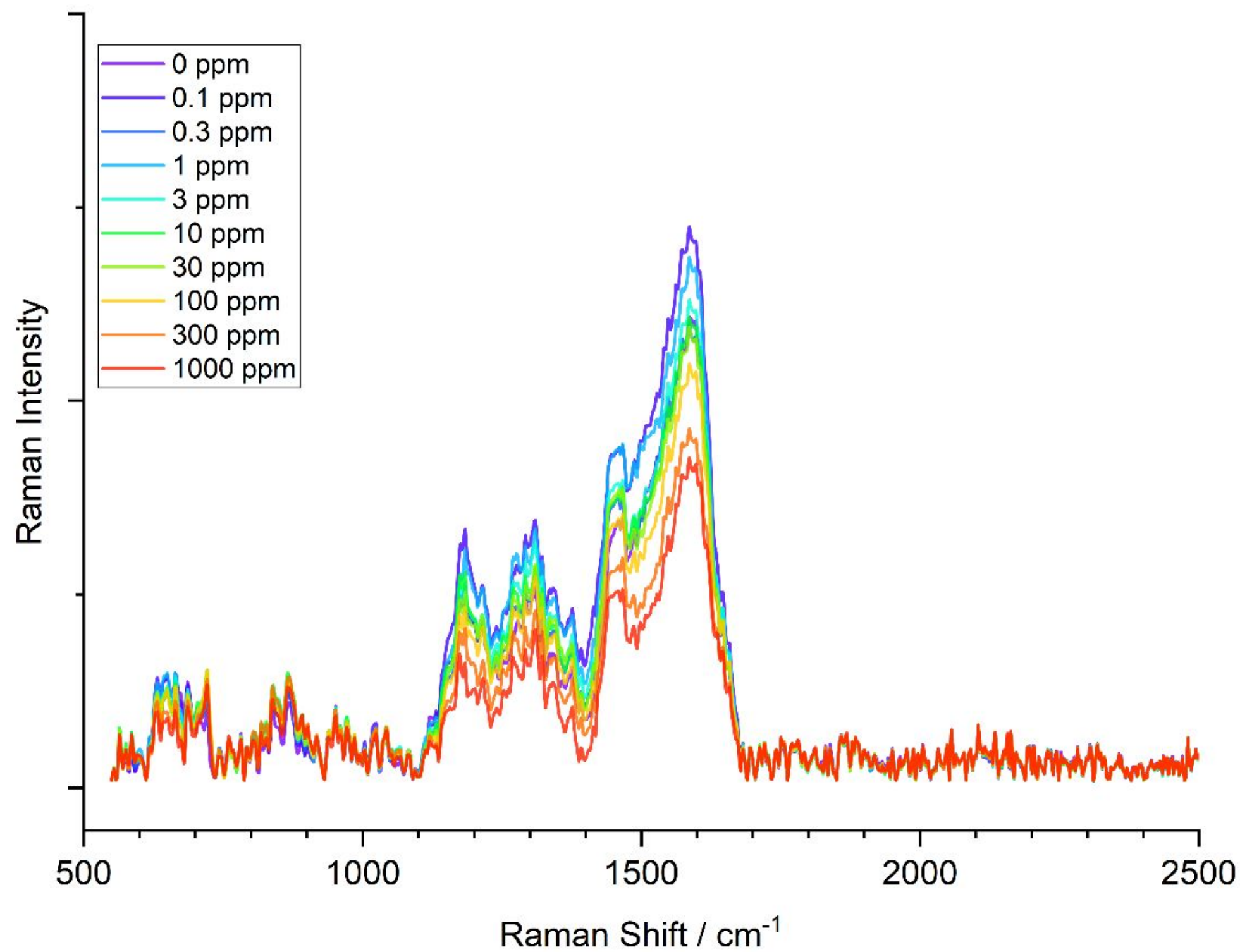

Figure S15: SERS spectra of GF dissolved in DCM at increasing concentrations.

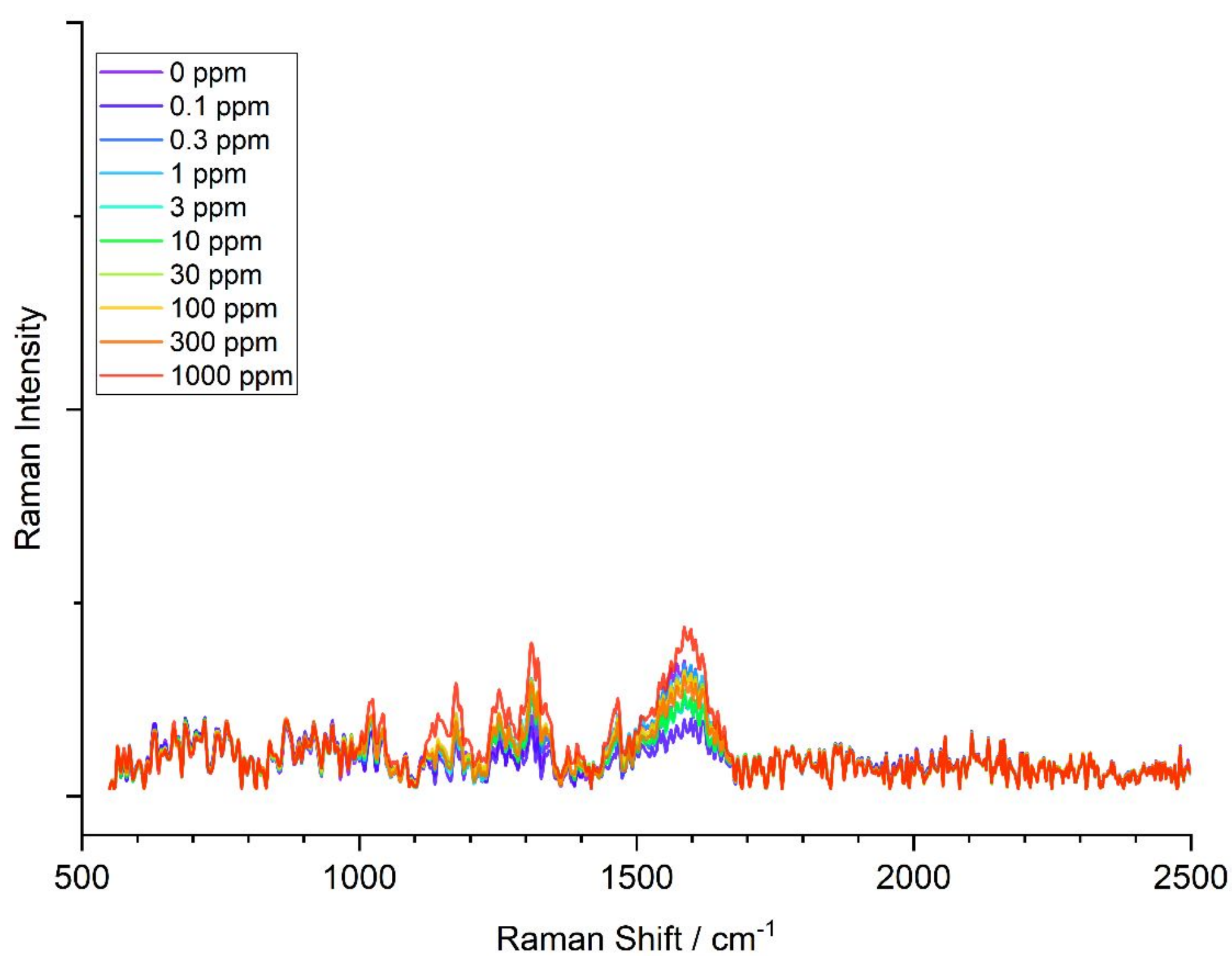

Figure S16: SERS spectra of GF dissolved in n-hexane at increasing concentrations.

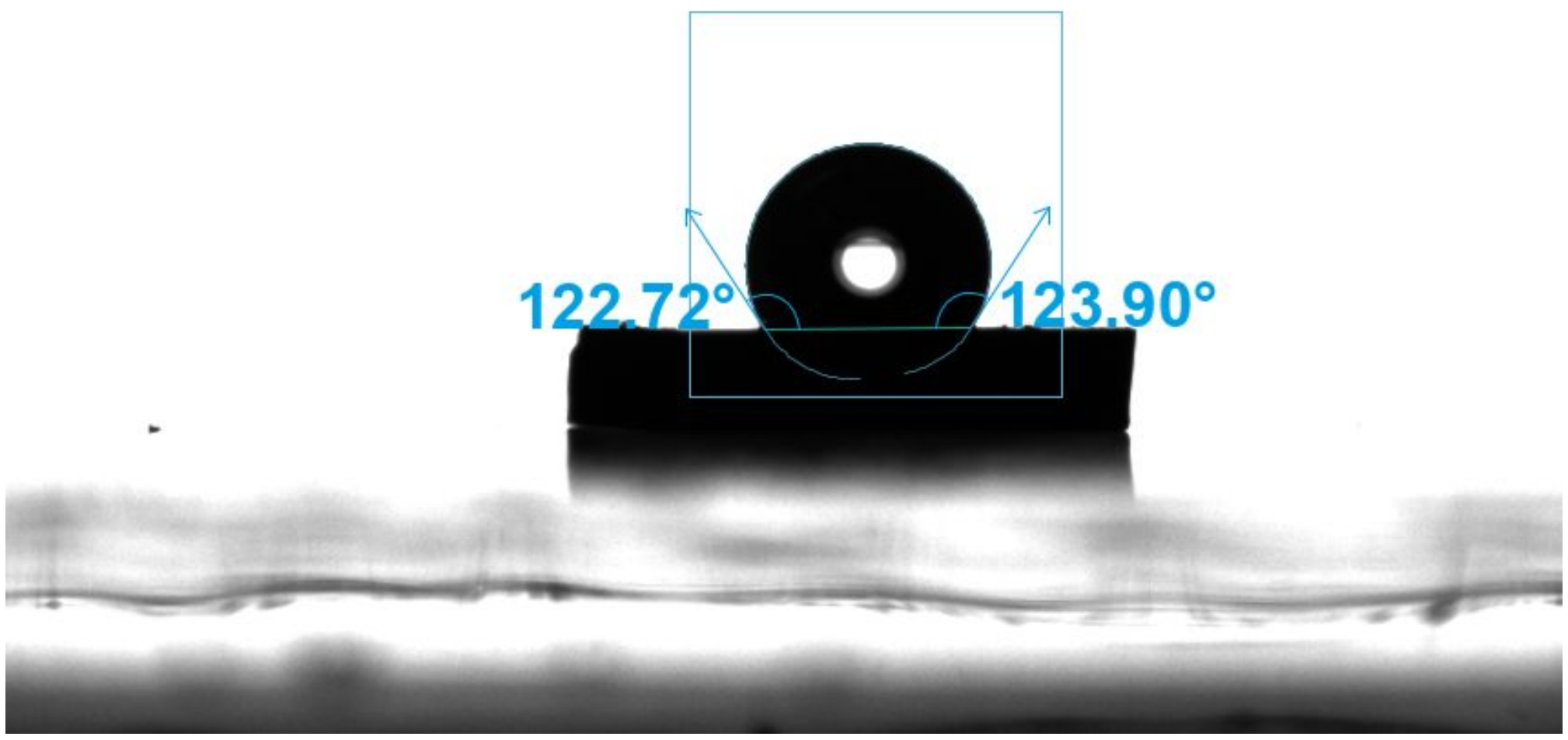

Figure S17: Contact angles of water droplet measured on gold nanopillar SERS substrate.

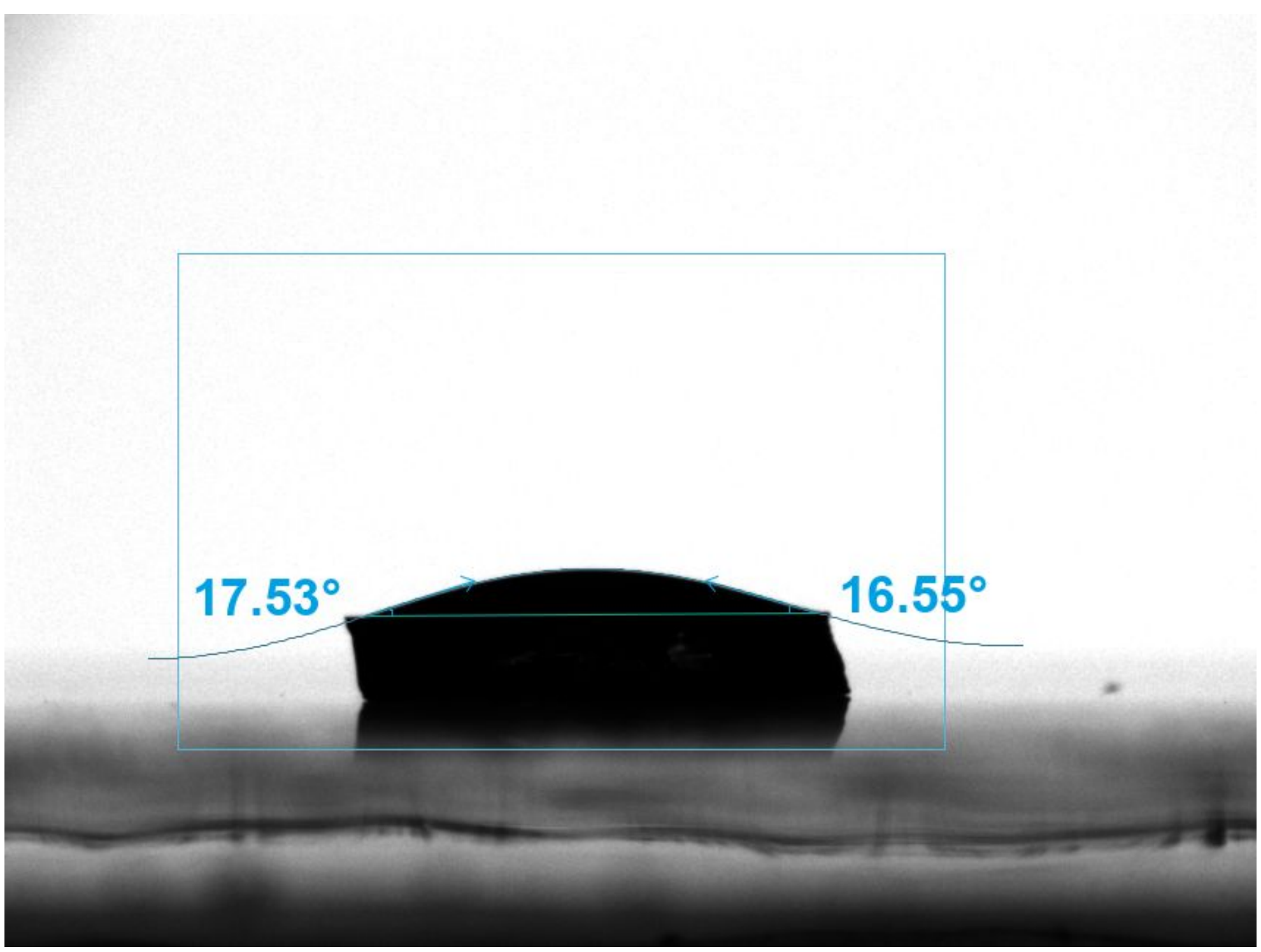

Figure S18: Contact angles of MeCN droplet measured on gold nanopillar SERS substrate. EtOH, DCM, and n-hexane displayed similarly low contact angles.

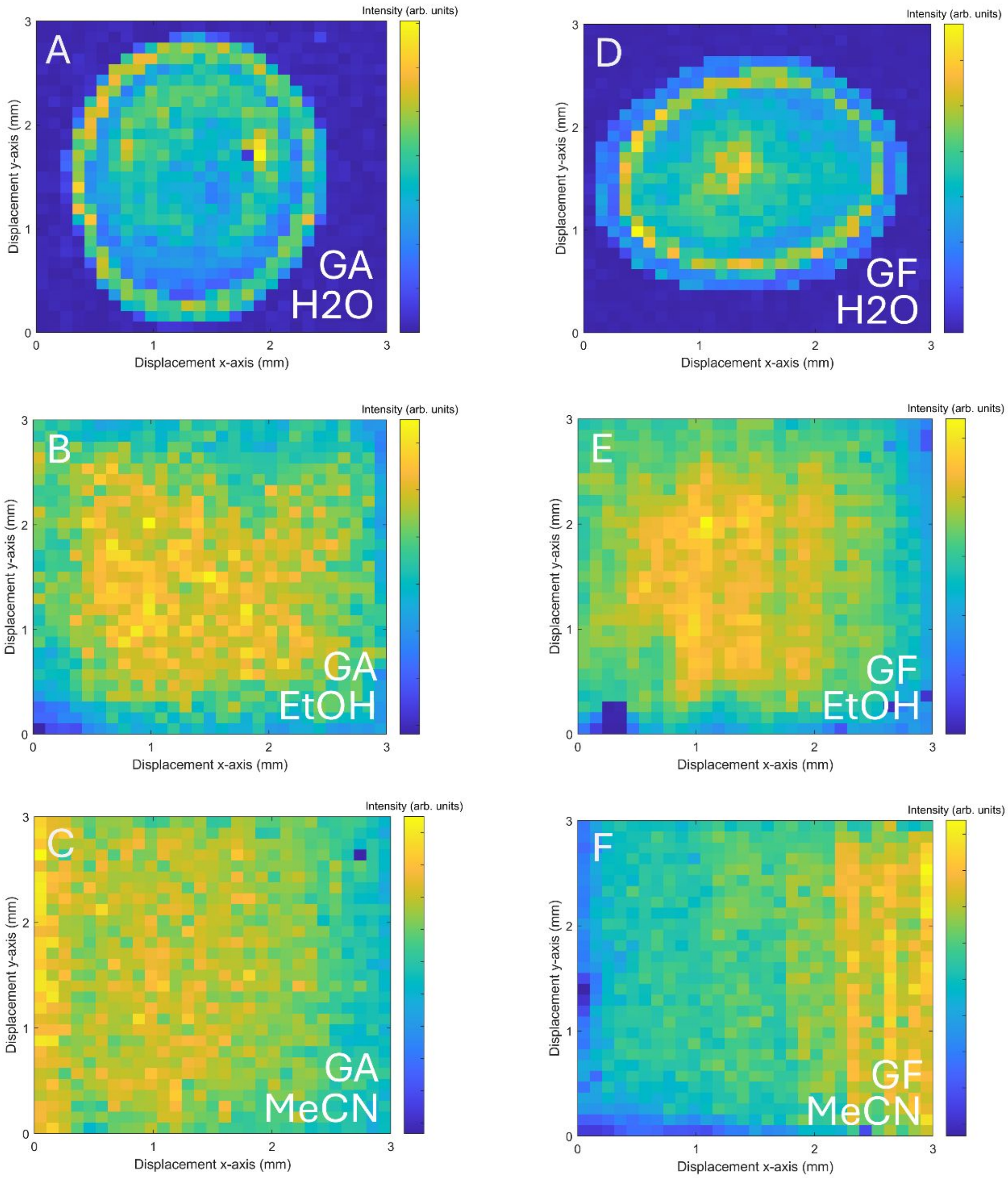

Figure S19: Raman maps of the 2150 cm^{-1} band of 30 ppm GA dissolved in H2O (A), EtOH (B), and MeCN (C), and the 760 cm^{-1} band of 300 ppm GF dissolved in H2O (D), EtOH (E), and MeCN (F).
